# An intermittent control model of flexible human gait using a stable manifold of saddle-type unstable limit cycle dynamics

**DOI:** 10.1098/rsif.2014.0958

**Published:** 2014-12-06

**Authors:** Chunjiang Fu, Yasuyuki Suzuki, Ken Kiyono, Pietro Morasso, Taishin Nomura

**Affiliations:** 1 Graduate School of Engineering Science, Osaka University, Toyonaka, Osaka, Japan; 2 Italian Institute of Technology, Genoa, Italy

**Keywords:** gait stability, intermittent control, bipedal locomotion, simulation, joint impedance, gait flexibility

## Abstract

Stability of human gait is the ability to maintain upright posture during walking against external perturbations. It is a complex process determined by a number of cross-related factors, including gait trajectory, joint impedance and neural control strategies. Here, we consider a control strategy that can achieve stable steady-state periodic gait while maintaining joint flexibility with the lowest possible joint impedance. To this end, we carried out a simulation study of a heel-toe footed biped model with hip, knee and ankle joints and a heavy head-arms-trunk element, working in the sagittal plane. For simplicity, the model assumes a periodic desired joint angle trajectory and joint torques generated by a set of feed-forward and proportional-derivative feedback controllers, whereby the joint impedance is parametrized by the feedback gains. We could show that a desired steady-state gait accompanied by the desired joint angle trajectory can be established as a stable limit cycle (LC) for the feedback controller with an appropriate set of large feedback gains. Moreover, as the feedback gains are decreased for lowering the joint stiffness, stability of the LC is lost only in a few dimensions, while leaving the remaining large number of dimensions quite stable: this means that the LC becomes saddle-type, with a low-dimensional unstable manifold and a high-dimensional stable manifold. Remarkably, the unstable manifold remains of low dimensionality even when the feedback gains are decreased far below the instability point. We then developed an intermittent neural feedback controller that is activated only for short periods of time at an optimal phase of each gait stride. We characterized the robustness of this design by showing that it can better stabilize the unstable LC with small feedback gains, leading to a flexible gait, and in particular we demonstrated that such an intermittent controller performs better if it drives the state point to the stable manifold, rather than directly to the LC. The proposed intermittent control strategy might have a high affinity for the inverted pendulum analogy of biped gait, providing a dynamic view of how the step-to-step transition from one pendular stance to the next can be achieved stably in a robust manner by a well-timed neural intervention that exploits the stable modes embedded in the unstable dynamics.

## 1. Introduction

Bipedal stability can be defined as the capacity to restore and maintain upright posture against external perturbations. It is of critical importance to human motor control during standing and walking in daily life [1]. Bruijn *et al.* [2] emphasized the importance of three requirements for achieving stability in the human gait system: (i) it should recover from small perturbations, such as tiny bumps on level ground and arm swing [3], which occur during every stride; (ii) it should be able to reorganize walking patterns in the case of large perturbations, for example, during stumbling [4], which requires changes in the gait trajectory; (iii) the largest perturbation recoverable by the system should be larger than the typical perturbations encountered in daily life. In this way, gait stability should always be measured by evaluating in which manner stable gait patterns are recovered in response to external perturbations. Here, we focus on the first requirement, namely on gait stability during steady-state periodic walking. This is the simplest requirement, on which the analysis of the two more general requirements can be built. However, in spite of the apparent simplicity, the fundamental mechanisms that allow this requirement to be fulfilled during human gait are still scarcely understood. This is primarily due to hierarchically distributed complexity of the neural centre of locomotion [5], and also due to the fact that elaborating neuro-muscular actuation patterns alone is not enough to predict whether or not they can realize stable mechanical gait motions of the body. Similarly, elaborating steady-state kinematics of a given gait alone is not enough to determine its degree of stability [6], because apparently identical gait kinematics can be accompanied with different degrees of stability.

Several measures to assess gait stability during steady-state walking have been proposed, including the maximum Floquet multiplier (FM) [7], the maximum Lyapunov exponent [8], the fractal exponents [9] and a set of nonlinear measures including approximate entropy for evaluating optimal motor variability [10], among others. These measures, which can be beneficial for evaluating the risk of falling in aged individuals and patients with motor dysfunctions, have been applied to the analysis of healthy and pathological gait. Despite this fact, however, there is no fully accepted quantitative way to judge the dynamic stability of human locomotion [11]. This is partially due to the fact that gait stability is determined by a number of cross-related factors, including gait trajectory, joint impedance and neural control strategies, and stability measures may vary a great deal depending on the underlying stabilization mechanisms. For example, gait variability does not necessarily imply low stability but it can be caused by enhanced internal noise [12]. Although it is generally agreed that the more rigidly a posture is stabilized, the smaller the postural variability, it is also clear that the typical rigidity (inflexibility) and related small postural sway in patients with Parkinson's disease is indeed the main cause of their postural instability [13]. Since the usual stability measures are obtained by non-parametric time-series analysis of experimental gait data, regardless of relevant underlying stabilization mechanisms, they are not suitable for understanding the causal relationship between values of a stability measure and underlying mechanisms that affect stability.

In this study, we take a model-based approach to consider stabilization mechanisms that can achieve steady-state periodic human gait, as in a number of previous related studies [6,14–16], but with more simple and analytical mind. Although some studies have been conducted with a similar approach, most of them analysed oversimplified models such as foot-less legged robots with or without active controllers [17–19], and thus the analytical results cannot be compared directly with human gait. We also consider a simplified model, but it is more anatomically plausible. We are particularly interested in the joint impedance that is characterized by stiffness and viscosity coefficients defined, respectively, by the first partial derivatives of torque with respect to joint angle and angular velocity [20]. A joint with a large impedance is said to be rigid, and that with a small impedance is flexible. It has been a common view that the brain stabilizes unstable body dynamics using impedance control, which resists destabilizing motion by increasing joint impedance [20,21]. We examine the contribution of the mechanical impedance of the leg joints to gait stability, in the assumption that joint impedance during physiological human gait is low. Indeed, Shamaei *et al.* have reported considerably small values of the quasi-stiffness (dynamic stiffness) of ankle (200–500 Nm rad^−1^ [22]), knee (200–350 Nm rad^−1^ [23]) and hip (200–600 Nm rad^−1^ [24]) joints during steady-state human gait with walking speed of about 1.4 m s^−1^. We demonstrate, later on, that any combination of joint stiffness in the range of values quoted above is far below the critical joint stiffness required for intrinsic gait stability, implying that physiological stabilization mechanism cannot simply be determined by conventional impedance control. Hence, we need to consider a control strategy that can achieve stable steady-state periodic gait while maintaining joint flexibility with the lowest possible joint impedance.

There is a number of possible candidates of motor strategies for stabilizing unstable dynamics, alternative to impedance control, for example, phase resetting control during biped gait [25,26], acceleration feedback control to compensate delay-induced postural instability [27] and intermittent feedback control that has been proposed for postural balancing during human quiet standing [28–33]. Although the phase resetting strategy is a powerful mechanism for improving gait stability [16,25], it is accompanied by modifications of the gait trajectory (phase-shift) with elevating and lowering patterns in response to external perturbations [4]. Thus, we characterize phase resetting as part of the above-mentioned requirement (ii), according to the classification proposed by Bruijn *et al.* [2]. In order to focus entirely on the stabilization mechanisms that do not determine any change in the trajectory, we exclude the phase resetting control from the current study. Moreover, effects of signal transmission delay in the neural feedback control, which can be critical for motor instability [27], are not considered in this study for simplicity. Nevertheless, later in this article, we will discuss complementary roles played by phase resetting to other strategies as well as effects of delay-induced instability on the neural control of bipedal gait.

The intermittent control paradigm has been considered for dynamics in the vicinity of steady-state behaviour of persistent actions such as human quiet standing [28,30,33] or tracking a target [34–36]. It exhibits *on-periods* and *off-periods* of time in activations of the feedback control torque. One of the related theories [28,30,33] claims that intermittent control is effective if a mechanical plant in the absence of active control exhibits saddle-type instability, as in the case of the inverted pendulum model of the standing human body. It is known indeed that during postural sway movements the body can transiently approach the upright position along a stable manifold of the unstable saddle-type equilibrium during each off-period. This means that transient converging saddle-type dynamics during the off-periods is primarily responsible for stabilizing the pendulum, supplemented by the active feedback control during short on-periods.

In this study, we extend the intermittent control paradigm, developed for quiet standing to steady-state human gait. In §2, we construct a heel-toe footed biped model with hip, knee and ankle joints and a heavy head-arms-trunk (HAT) element in the sagittal plane. The model uses a motion-captured periodic profile of joint angles from a healthy young adult as a desired or reference trajectory to be tracked by a proportional-derivative (PD) feedback controller, whereby the joint impedance is parametrized by the PD-gains. A preliminary work is performed, prior to carrying out the forward dynamic simulations of the model, in order to identify a desired steady-state gait, i.e. a HAT motion that cannot be specified by the desired joint angle trajectory, and its associated ground reaction force (GRF) profile, that are consistent with the equation of motion. This preliminary task allows us to construct a feed-forward controller for the desired gait and to isolate only the joint impedance-related stability issue from the cross-related factors. More specifically, the motion-trajectory-dependency of joint impedance is limited and fixed by considering the kinematically identical walking according to the desired gait, regardless its degree of stability for any given values of the PD-gains.

After introducing methodological definitions in §3, we show in §4 that the desired gait can be established as a stable limit cycle (LC) for an appropriate set of impedance controllers with large PD-gains. Since the feedback and feed-forward torques are time periodic, and thus the equation of motion of the model can be formulated as a non-autonomous periodically forced nonlinear dynamical system, stability change of the LC as a function of the PD-gains are analysed using Floquet theory. This analysis can be performed easily because the solution for LC has already been specified as the desired gait for any values of the PD-gains. We then show that, as the PD-gains decrease for lowering the joint stiffness, stability of the LC is lost only in a few dimensions, while leaving the remaining large number of dimensions quite stable, by which the LC can be classified as a saddle-type instability, with a low-dimensional unstable manifold and a high-dimensional stable manifold.

Finally, in §5, we propose an intermittent neural feedback controller that is activated only for a short period of time at an optimal phase of each gait stride. We show that intermittent control paradigms can stabilize the unstable LC in a more robust way, with very small values of the PD-gains, in a consistently biomimetic manner. We discuss physiological plausibility of the intermittent control and relations to other control strategies in §6.

## 2. A model of biped gait

**Figure 1.**
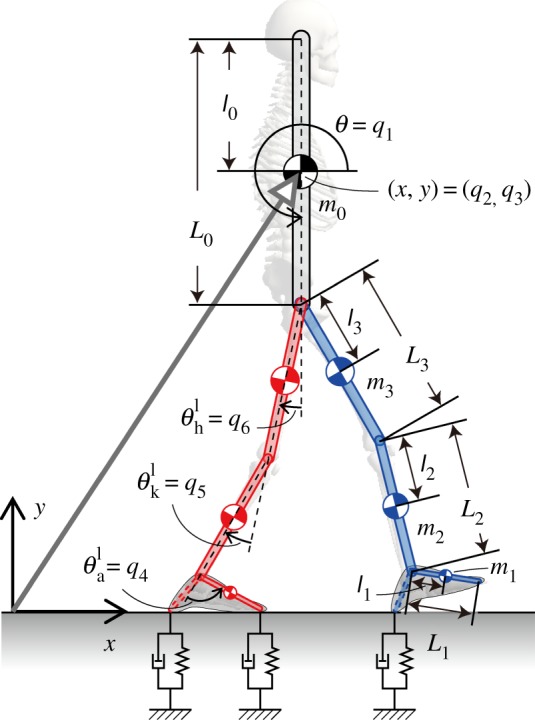
A model of biped gait, which operates in the sagittal plane and includes seven links (HAT, left and right feet, shanks and thighs) with six joints (hip, knee and ankle joints of both legs). A posture of the model is specified by the general coordinate *q* = (*q*_1_, … , *q*_9_)^T^. *θ* = *q*_1_ and (*x*, *y*) = (*q*_2_, *q*_3_) represent the tilt angle and the position of HAT-CoM, respectively. Ankle, knee and hip joint angles are represented by (*q*_4_, *q*_5_, *q*_6_) for the left leg and (*q*_7_, *q*_8_, *q*_9_) for the right leg. They are also denoted by 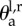, 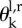 and 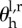. Masses of foot *m*_1_, shank *m*_2_, thigh *m*_3_ and HAT *m*_0_ are 0.682, 3.162, 6.882 and 40.548 kg, respectively. *L*_1_ = 0.122 m, *L*_2_ = 0.379 m, *L*_3_ = 0.420 m, and *L*_0_ = 0.536 m. See table 1 for the complete set of parameter values. Vertical and horizontal GRFs are modelled by nonlinear spring-damper systems. See the electronic supplementary material A for the equations of motion and the model of GRFs.

A heel-toe footed biped gait model constructed by Yamasaki *et al.* [25] is used in this study (figure 1). It operates in the sagittal plane and includes seven links (HAT, left and right feet, shanks and thighs) and six joints (hip, knee and ankle joints of both legs). A posture of the model can be specified uniquely by2.1
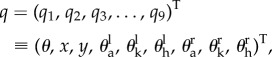
where the elements of the vector, from the first to the ninth, are the HAT tilt angle, horizontal and vertical positions of the HAT centre of mass (CoM), ankle, knee, hip joint angles of the left limb and those of the right limb. Yamasaki *et al*. performed forward dynamic simulations with constraining all of the six joint angles by an experimentally motion-captured kinematic data using a method developed by Van Den Bogert *et al.* [37]. Specifically, the joint angles are constrained as2.2
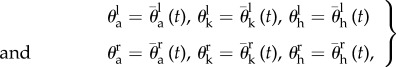
where 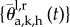 is a set of motion-captured periodic joint angles with period *T* = 1.135 s, from a healthy young subject during steady-state walking. Each of those angles was fitted by the eighth order of Fourier series, and all coefficients are available in [25]. In this way, the dimension of the model's state vector is reduced from 18 to six, namely the HAT variables (*q*_1_ = *θ*, *q*_2_ = *x*, *q*_3_ = *y*) and the corresponding time derivatives. This means that walking of the model is driven only by the GRFs that are produced by nonlinear interactions between the ground and the wallowing multi-link model. See figure 1 and the electronic supplementary material A for the equations of motion and the model of GRFs. The crucial feature of this model, referred to here as *the constraint model*, is that it exhibits a stable periodic gait without any feed-forward and feedback controllers, if initial conditions are set appropriately within a certain area of the state space, as shown by Yamasaki *et al.* [25]. It is important to note that this fact is solely responsible for the following stability analysis of the non-constraint model without the joint constraint. In other words, an optimal set of joint angle kinematics has been selected specifically for the model, and other joint angle trajectories largely different from the one used in this study may not achieve stable walking of the constraint model. This means that gait trajectory (joint angle trajectory in this context) is also a very important determinant of gait stability (see Discussion.) Here, we denote the steady-state periodic motion achieved by the constraint model, in terms of *q*, as2.3
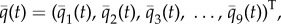
where 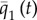, 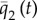 and 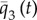 are the steady-state periodic solution of the constraint model, and the remaining 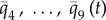 are just the periodic kinematic data defined in equation (2.2). Thus, 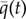 is a periodic function of time with gait period *T*, i.e. 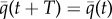.

In this study, we release the constraint on the joint angles, which brings the degree of freedom and the dimension of the model's state space back to nine and 18, respectively. Because no kinematic constraint is posed on the joint angles, we need to introduce joint torque actuations to realize a periodic steady-state gait of the non-constraint model, whose equations of motion can then be represented as2.4
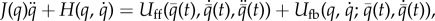
where2.5
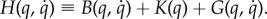
*J* is the 9 × 9 inertia matrix, and *B*, *K* and *G* are nine-dimensional vectors of centrifugal and Coriolis torque, gravitational torque and GRF, respectively. See table 1 for body parameters and the electronic supplementary material A for details of *J* = (*j_i,k_*), *B* = (*b_i_*), *K* = (*k_i_*) and *G* = (*g_i_*) for *i*, *k* = 1, 2, … , 9. *U*_ff_ and *U*_fb_ are the feed-forward and feedback control torques, respectively. In this way, equation (2.4) describes the motion of the rigid-link model during tracking the periodic desired joint angle trajectory. We assume that the feed-forward controller uses a precise inverse dynamics model defined as2.6
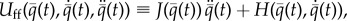
and the feedback controller is simply based on a PD feedback mechanism defined by2.7
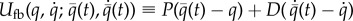
with *P* and *D* being the following proportional and derivative gain matrices2.2
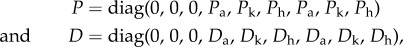
where *P*_a_–*D*_a_, *P*_k_–*D*_k_ and *P*_h_–*D*_h_ are the gains of PD controllers acting on the ankle, knee and hip joints, respectively. Note that, since the first three elements of *P* and *D* gain matrices are zero, the feedback control torque *U*_fb_ is determined only by the joint angles (*q*_4_, *q*_5_, *q*_6_, *q*_7_, *q*_8_, *q*_9_) and 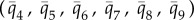, i.e. independent of (*q*_1_, *q*_2_, *q*_3_), although equation (2.7) in its appearance is written as the function of the whole *q* and 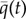. Note also that the choice of the diagonal matrices for *P* and *D* gains are just for mathematical simplicity, and they can include non-diagonal elements in reality.

**Table 1.** Values of the body parameters used in the biped model.

symbol	description	
(*x*, *y*)	*x*–*y* positions of HAT-CoM (*q*_2_ and *q*_3_ in the general coordinate)	
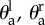	ankle joint angle (*q*_4_ and *q*_7_ in the general coordinate)	
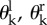	knee joint angle (*q*_5_ and *q*_8_ in the general coordinate)	
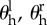	hip joint angle (*q*_6_ and *q*_9_ in the general coordinate)	
*θ*	posture of HAT (*q*_1_ in the general coordinate)	
*g*	gravitational acceleration	9.8 (m s^−2^)
*m*_1_	mass of foot	0.682 (kg)
*m*_2_	mass of shank	3.162 (kg)
*m*_3_	mass of thigh	6.882 (kg)
*m*_0_	mass of HAT	40.548 (kg)
*L*_1_	length from ankle joint to toe	0.122 (m)
*L*_2_	length of shank	0.379 (m)
*L*_3_	length of thigh	0.420 (m)
*L*_0_	length of HAT	0.536 (m)
*l*_1_	distance from ankle joint to CoM of foot	0.050 (m)
*l*_2_	distance from ankle joint to CoM of shank	0.154 (m)
*l*_3_	distance from ankle joint to CoM of thigh	0.200 (m)
*l*_0_	distance from ankle joint to CoM of HAT	0.332 (m)
*I*_1_	inertia moment of foot	0.00014 (kg m^2^)
*I*_2_	inertia moment of shank	0.03001 (kg m^2^)
*I*_3_	inertia moment of thigh	0.09485 (kg m^2^)
*I*_0_	inertia moment of HAT	1.09933 (kg m^2^)

It is important to note that 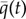 defined by equation (2.3) is *always* a solution of equation (2.4), because the following equality holds, regardless of the PD-gain values:2.8
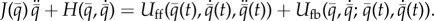
The reason is threefold: (i) 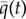 is the steady-state solution of the constraint model, (ii) *U*_ff_ is a precise inverse dynamics solution of the constraint model, and (iii) 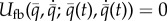 in the right-hand side of equation (2.8), although stability of the solution 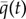 depends on the PD-gains. This means that, when we analyse stability of the model for a given set of PD-gains, we do not need to search a periodic solution of equation (2.4), which is usually a quite time-consuming task, but we can just analyse stability of the solution 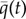.

A state space representation of equation (2.4) is as follows:2.9
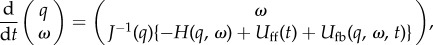
where 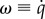,
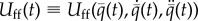
and
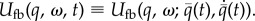


Moreover, by defining 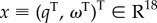, equation (2.9) can be written as2.10
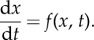


As 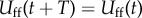 and *U*_fb_(*x*, *t* + *T*) = *U*_fb_(*x*, *t*), we have2.11
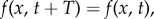
meaning that equation (2.9) (equation (2.10)) is a periodically forced mechanical system, which is also a non-autonomous dynamical system with a *T*-periodic vector field.

Once again, since the feed-forward controller *U*_ff_(*t*) outputs the precise inverse dynamic solution of the rigid-link model, equation (2.10) *always* possesses a periodic solution that corresponds to 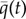. We denote it by2.12
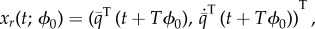
for which 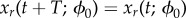, where *ϕ*_0_ represents the initial phase of the periodic function defined by equation (2.3). The explicit representation of *ϕ*_0_-dependency is useful for a later purpose. The closed trajectory of *x_r_*(*t*;*ϕ*_0_) forms an LC attractor in the phase space of R^18^. We are interested in the stability of LC.

An initial condition at *t* = 0 for solving the initial value problem of equation (2.10) should be formulated with a special care on *the initial phase of the desired trajectory*, *ϕ*_0_. That is, for a given initial state *x*(0), there is a freedom of choice for *ϕ*_0_. In other words, a solution of equation (2.10) starting from *x*(0) with an initial phase of the desired trajectory *ϕ*_0_ of *x_r_*(0; *ϕ*_0_) and that with another initial phase of the desired trajectory 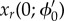 are not the same. The difference between those two solutions are not necessarily only the phase of the steady-state oscillation (*ϕ*_0_ versus 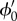), but it can also happen that one solution asymptotes to the desired trajectory and the other solution does not. This is associated with a basin of attraction of LC [25].

## 3. Stability and joint impedance

This section summarizes briefly how we evaluate stability of the LC for the biped model. We also provide a formal definition of joint impedance for the biped model and a mathematical formulation of how stability of the LC can be associated with joint impedance.

### 3.1. Floquet stability

Consider a state point of the system 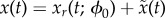 with 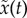 being a small perturbation (error state) vector from the LC. In Floquet theory, stability of the LC is determined by the time evolution of 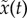, which is described by3.1
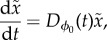
where the Jacobian matrix 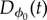 is *T*-periodic function of *t*3.2
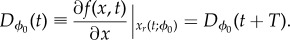
Since equation (3.1) possesses *n*(=18) linearly independent solutions for a set of *n* linearly independent initial conditions 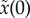, we consider3.3
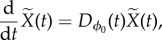
where 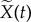 is composed of the *n* linearly independent column vectors. 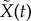 can be obtained by integrating equation (3.3) from *t* = 0 with an initial phase *ϕ*_0_ of the periodic desired trajectory 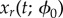. The *n* × *n* identity matrix *I* is usually chosen as an initial error state matrix 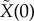. The solution integrated over the cycle, 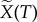, is referred to as the monodromy matrix 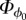. Note that the monodromy matrix is represented differently depending on the initial phase *ϕ*_0_.

FMs, denoted by *λ_i_* (*i* = 1, … , *n*), are defined as the eigenvalues of 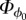. If 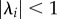 for *i* = 1, … , *n*, the perturbation 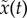 decays to zero, meaning that the LC is asymptotically stable, and unstable otherwise. Stroboscopic observations of the error state 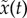 at discrete instants of time *t* = 0, *T*, 2*T*, 3*T*, etc., represent the cycle stability of the LC.

We obtained the Jacobian 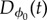 by numerically differentiating *f*(*x*, *t*) of equation (2.10), and then evaluating it along *x_r_*(*t*; *ϕ*_0_) through the gait cycle 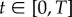. We then examined how stability of the LC changes depending on the values of *P*-gains. See the electronic supplementary material B for validation of the numerical evaluation of the Jacobian.

### 3.2. Joint impedance as a stability determinant

Joint impedance is characterized by stiffness matrix *K_d_* and viscosity matrix *B_d_*, which are usually defined, respectively, by the derivatives of the total joint torque with respect to the position and the velocity. They are equal to *P* and *D* in our biped model as follows:3.4
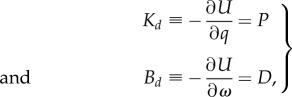
where 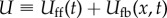. Thus, we considered simply the PD-gains of the feedback controller as the joint impedance in this study. This means that the smaller the values of PD-gains (impedance), the more flexible are the corresponding joints.

It is also worthwhile to consider a different type of joint impedance, which is more directly related to Floquet stability of the LC, 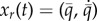. Considering a perturbed solution of equation (2.10) as 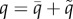, 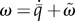, 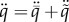, we have the following linearized equation, which describes dynamic evolution of the perturbation:3.5
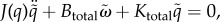
where3.6
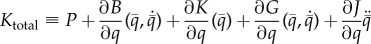
and3.7
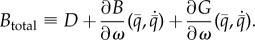
Note that, in equation (3.6), we use the following notation:
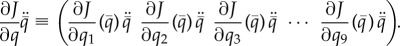


Furthermore, *K*_total_ and *B*_total_ can be conveniently related to the Jacobian matrix 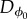 around the LC as follows:3.8
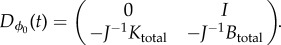


In this way, the total joint impedance, which is characterized by *K*_total_ and *B*_total_, determines values of FMs. See the electronic supplementary material C for the derivation of equations (3.6)–(3.8). The fact that *K*_total_ and *B*_total_ are the functions of not only *P* and *D* but also 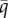 and 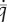 implies that joint kinematics (i.e. gait kinematics) is also an important determinant of joint impedance, thus joint flexibility and gait stability, although it is given and fixed as 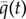 in this study.

## 4. Simulations and numerical analysis

We performed numerical simulations of the model by integrating equation (3.6) and/or equation (3.3) simply using the forward Euler method with a time step of Δ*t* = 10*^−^*^5^ s, where the use of this Δ*t* was validated by confirming that simulations with 10 times larger and 10 times smaller values of Δ*t* did not alter dynamics of the model. For each simulation, an initial condition *x*(0), i.e. an initial state of the body, and an initial phase *ϕ*_0_ were specified, in which *x*(0) was set close to the LC such that 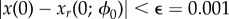 was satisfied for a given *ϕ*_0_. This means that the model was already in the middle of walking at the beginning of every simulation, and our model cannot deal with a gait initiation from quiet standing. In this article, for simplicity, simulation results with the following single initial state are shown; 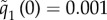 rad, 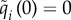 for *i* = 2, 3, … , 9 and 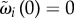 for *i* = 1, 2, … , 9. However, we confirmed that changes in the initial state did not alter the results shown below as far as 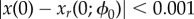 was satisfied.

Since stability of the LC depends on the values of PD-gains, each of *P*_a_, *P*_k_ and *P*_h_ were varied between 0 and 1500 Nm rad^−1^. *D*_a_, *D*_k_ and *D*_h_ were fixed at a quite small value (10 Nms rad^−1^) throughout the study.

**Figure 2.**
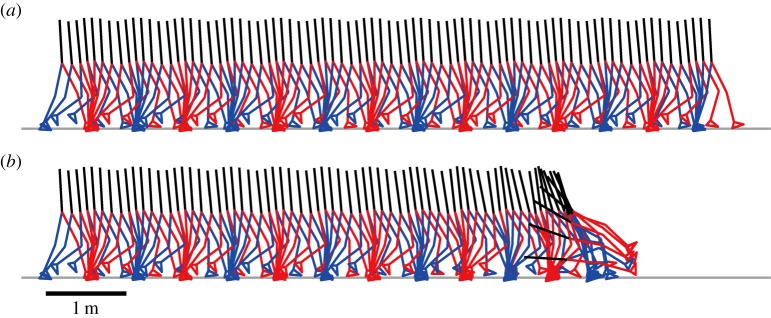
Stick pictures of a stable gait ((*a*) for large *P*-gains; *P*_a_ = *P*_k_ = *P*_h_ = 1500 Nm rad^−1^) and an unstable gait ((*b*) for small *P*-gains; *P*_a_ = *P*_k_ = *P*_h_ = 700 Nm rad^−1^) of the model obtained by numerical simulations of equation (2.10). For both cases, initial perturbations were set as 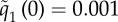 rad, 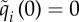 for *i* = 2, 3, … , 9 and 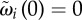 for *i* = 1, 2, … , 9, i.e. only the HAT tilt angle component 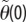 was perturbed from the LC.

Figure 2 exemplifies stable and unstable gait patterns obtained for large and small *P*-gains, respectively. (*P*_a_ = *P*_k_ = *P*_h_ = 1500 Nm rad^−1^ for the stable case, and *P*_a_ = *P*_k_ = *P*_h_ = 700 Nm rad^−1^ for the unstable case.) We verified that dynamics of the model during stable gait was almost exactly the same as that of the constraint model for various examined sets of large values of *P*-gains, confirming that steady-state walking of the constraint model, 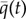 in equation (2.3), always forms the LC of the unconstraint model, i.e. equation (2.10). This should also be true for small *P*-gains, although numerical confirmation (using shooting method, for example) is not easy because the LC is unstable for such cases.

**Figure 3.**
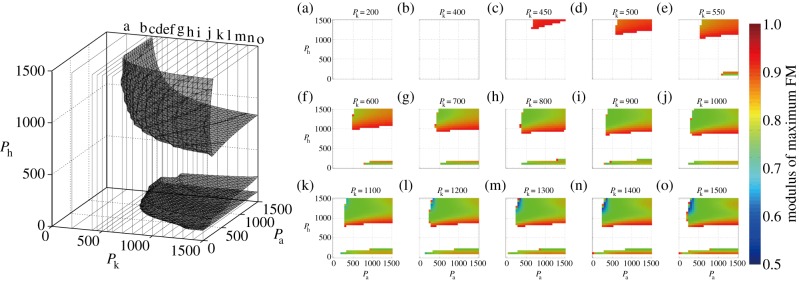
Asymptotic stability regions (stability boundary) in the *P*_a_*−P*_k_*−P*_h_ parameter space (left panel) and distribution of the maximum FM within the stable regions (panels on the right). These panels (indexed as eaf, ebf, c, eof) are the *P*_a_*−P*_h_ cross-sections of the *P*_a_*−P*_k_*−P*_h_ parameter space for different values of *P*_k_. In such cross-sections, the modulus of the maximum FM of the monodromy matrix 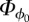 is colour-coded as indicated by the vertical colour-code bar in the right-most of the figure: white means instability with maximum FM larger than 1, red means close to instability with maximum FM near 1 and blue means good stability with the maximum FM far below 1 (near 0.5). Measurement unit of *P*_a_*−P*_k_*−P*_h_ parameter: Nm rad^−1^.

Figure 3 displays the stability regions and distribution of the maximum FM(s) within the stability regions in the *P*_a_−*P*_k_−*P*_h_ parameter space: in particular, it clearly shows that the main stability region is associated with large values of *P*-gains. Roughly speaking, for stability of the LC in the main stability region, *P*_h_ should always be larger than 750 Nm rad^−1^, implying the importance of high stiffness of the hip joints for stability, whereas either *P*_a_ or *P*_k_ can have smaller values. For example, if we consider cases with small *P*_a_ values of about 200–300 Nm rad^−1^, *P*_k_ should be larger than 1000 Nm rad^−1^: this means that a flexible ankle joint should accompany a stiff knee joint. On the other hand, the minimum value of *P*_k_ is about 450 Nm rad^−1^, which should be accompanied by *P*_a_ and *P*_h_ larger than 500 and 1000 Nm rad^−1^, respectively. Thus, the qualitative picture for achieving gait stability can be summarized as follows: the hip joint should be rather stiff, but the ankle joint can be flexible; the knee joint should be very stiff with flexible ankles, but can have medium values otherwise.

Figure 3 also shows that there is a smaller stability region for small values of *P*_h_ ∼ 100 Nm rad^−1^. This is an opposite situation to the main stability region, suggesting that the hip joint must not be necessarily stiff for gait stability. For this thin region, typical combinations of *P*_a_ and *P*_k_ that can stabilize the LC are located at (*P*_a_, *P*_k_, *P*_h_) ∼ (1200, 550, 100) for the most flexible knee joint case with very stiff ankle joints, (*P*_a_, *P*_k_, *P*_h_) ∼ (500, 700, 100) and (*P*_a_, *P*_k_, *P*_h_) ∼ (30, 1500, 100) for the most flexible ankle joint case with very stiff knee joints.

Quantitative examination of the distribution of the maximum FM(s) within the stability regions reveals that the optimal combination of *P*-gains for the highest stability within the examined range is located at (*P*_a_, *P*_k_, *P*_h_) ∼ (300, 1400, 1300). This means that the largest stiffness case examined in this study, i.e. *P*_a_ = *P*_k_ = *P*_h_ = 1500 Nm rad^−1^ does not provide the highest stability of the LC, and gait stability does not increase simply as the joint impedance (stiffness) increases.

**Figure 4.**
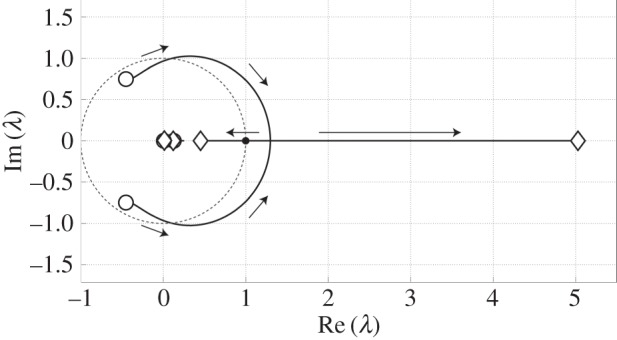
Root loci of FMs when the *P*-gains decrease as (*P*_a_, *P*_k_, *P*_h_) = (1500, 1500, 1500) − *p·*(1, 1, 1) for 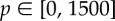. The FMs indicated by open circles and diamonds are for (*P*_a_, *P*_k_, *P*_h_) = (1500, 1500, 1500) and (0, 0, 0), respectively. The black circle always stays at (1,0) for any values of *P*-gains, and it represents the linear increase of the horizontal HAT-CoM position variable *q*_2_(=*x*).

Figure 4 shows the root loci (loci of FMs) when the *P*-gains decrease according to the following law (*P*_a_, *P*_k_, *P*_h_) = (1500, 1500, 1500) − *p·*(1, 1, 1), with the scalar parameter *p* changing smoothly from 0 to 1500. We can observe that all FMs, except one FM of unity at (1, 0) on the unit circle, are located within the unit circle on the complex plane for large values of *P*-gains. For example, see the FMs indicated by open circles for (*P*_a_, *P*_k_, *P*_h_) = (1500, 1500, 1500) in figure 4. This means that the LC is stable for large *P*-gains, because one unity FM reflects the fact that the variable *q*_2_(=*x*) representing the horizontal position of the HAT-CoM grows linearly (with a constant ratio) for the cyclic observations as the model continues walking in the direction of *q*_2_(=*x*) on the ground. Thus, we do not always take into account this unity FM for determining gait stability and the maximum FM. (This is also the case for obtaining figure 3.) As the parameter *p* increases, thus reducing the (*P*_a_, *P*_k_, *P*_h_) values, one pair of complex conjugate FMs crosses the unit circle at *P*_a_ = *P*_k_ = *P*_h_ ∼ 890 Nm rad^−1^, where stability of the LC is lost (gait instability). This type of instability is known as the Hopf (Neimark–Sacker) bifurcation [38], which may lead to quasi-periodic dynamics. However, we could verify that at this bifurcation not only the local stability but also the global stability of LC is lost, leading to a fall of the model. Thus, we never observe quasi-periodic dynamics in our model. Note that, as we mentioned earlier, the LC defined by equation (2.12) exists persistently as an unstable LC for small values of the *P*-gains even after the instability.

As the *P*-gains decrease further, the loci for the pair of complex conjugate FMs, which are the dominant mode of the dynamics, collide with each other, and then becomes two distinct real FMs at *P*_a_ = *P*_k_ = *P*_h_ ∼ 645 Nm rad^−1^; with a further decrease, one of the real FM moves left and the other goes right in the complex plane, namely the former FM returns back into the unit cycle for *P*_a_ = *P*_k_ = *P*_h_ ∼ 639 Nm rad^−1^, after which only one real FM remains as the unstable mode. The degree of instability of this mode increases as the *P*-gain decreases, whereas the remaining modes remain quite stable.

### 4.1. Stable and unstable manifolds of the limit cycle

Although the FMs are independent of the initial phase *ϕ*_0_ because of the periodic nature of the LC, the eigenvectors are *ϕ*_0_-dependent as is 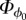. The matrix 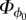 can be diagonalized as4.1
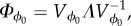
where 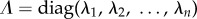 with *n* = 18, which is *ϕ*_0_-independent, and4.2
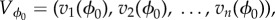
with 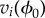 being the *ϕ*_0_-dependent eigenvector of 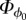 for *λ_i_*. For the diagonalization of 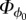, we prefer to use the Jordan normal form. Thus, for a pair of complex conjugate FMs *λ_i_* and 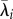, we employ two real basis vectors 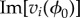 and 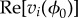, instead of 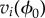 and 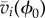. Then the corresponding diagonal 2 × 2 block of the matrix 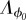 becomes4.3
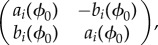
where 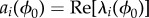 and 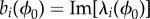.

**Figure 5.**
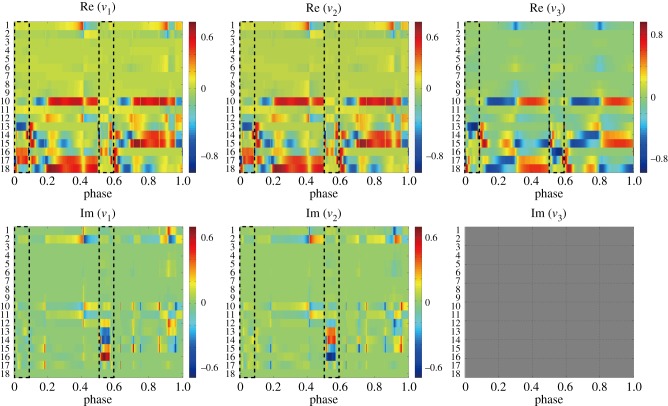
Initial phase *ϕ*_0_-dependency of eigenvectors of the three dominant FMs of 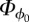. In this example, *P*_a_ = *P*_k_ = *P*_h_ = 700 Nm rad^−1^, for which the LC is unstable, and the dominant FMs, except the single unity FM, are a pair of complex conjugates *λ*_1_ and 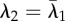 with 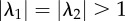 and one real *λ*_3_ < 1, for which eigenvectors are *v*_1_(*ϕ*_0_), 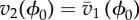 and 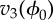, respectively. In each panel, 18 element-values of the vectors 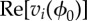 and 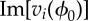 are colour-coded as indicated by the vertical colour-code bar in the right-most of each panel. The phase origin corresponds to the left heel-contact throughout the paper. Dotted squares in each panel indicate the double support phases. One can observe that each eigenvector changes in a continuous manner basically as the function of *ϕ*_0_, but it exhibits abrupt changes at the heel-contact and toe-off events.

Figure 5 exemplifies how the eigenvectors of the three dominant FMs (except one unity FM) change as functions of the initial phase *ϕ*_0_ from which equation (3.3) was integrated to obtain the monodromy matrix 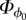. In this example, *P*_a_ = *P*_k_ = *P*_h_ = 700 Nm rad^−1^, for which the LC is unstable, and the dominant FMs, except the one unity FM, are a pair of complex conjugates *λ*_1_ and 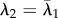 with 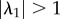 and one real FM with 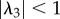. Note that, in this case, the remaining 14 FMs are all located within the unit cycle, and their moduli are much smaller than 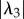. One can observe that each eigenvector of the monodromy matrix 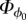 changes in a continuous manner basically as the function of *ϕ*_0_, but it exhibits abrupt changes at the heel-contact and toe-off events. This means that the direction of local convergent flow towards the LC and that of local divergent flow away from the LC change as the function of *ϕ*_0_ along the LC.

We are interested in how an initial error state 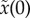 evolves in the cycle-to-cycle basis. The matrix 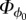 describes such an error state evolution based on the sequence of *T*-periodic stroboscopic observations of 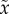 when the phase of desired trajectory becomes 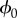 periodically, which is described as4.4
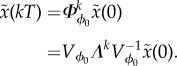
with *k* counting the cycle number of gait.

This is a linear discrete dynamical system defined by the map 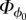. Let us consider dynamics of equation (4.4) more in detail using the case shown in figure 5. We consider only the three dominant FMs (*λ*_1_, 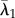 and *λ*_3_), for simplicity. Denoting 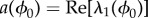 and 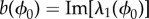, we have an analytical solution of equation (4.4) with respect to the eigenvector basis as follows:4.5
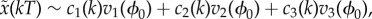
where 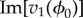 and 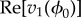 of the complex eigenvector 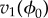 for *λ*_1_ are rewritten as *v*_1_(*ϕ*_0_) and *v*_2_(*ϕ*_0_), respectively, and *v*_3_(*ϕ*_0_) is the real eigenvector for *λ*_3_. The coefficients *c*_1_, *c*_2_ and *c*_3_ are given by4.6
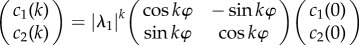
and4.7
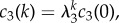
where 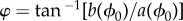. Note that the coefficients *c*_1_(*k*) and *c*_2_(*k*) represent the error state dynamics projected on the two-dimensional subspace spanned by *v*_1_(*ϕ*_0_) and *v*_2_(*ϕ*_0_) associated with the unstable FMs (*λ*_1_ and 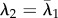), and 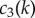 represents those on the one-dimensional subspace spanned by *v*_3_(*ϕ*_0_) associated with the stable FM (*λ*_3_). Thus, *c*_1_(*k*) and *c*_2_(*k*) diverge according to equation (4.6), but *c*_3_(*k*) converges to zero, as far as 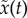 is small and the linear approximation is valid, according to equation (4.7).

**Figure 6.**
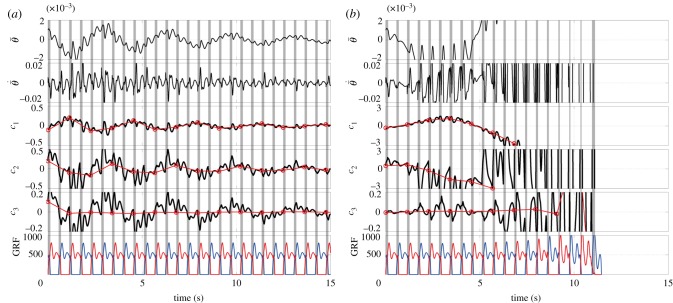
Stable and unstable gait dynamics with large ((*a*); *P*_a_ = *P*_k_ = *P*_h_ = 1500 Nm rad^−1^) and small ((*b*); *P*_a_ = *P*_k_ = *P*_h_ = 700 Nm rad^−1^) feedback gains. 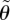 and 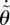 represent the error states of the tilt angle (rad) and angular velocity (rad s^−1^) of HAT. Panels (*a*) and (*b*) correspond to the upper and lower panels of figure 2, respectively. The coefficients *c*_1_(*k*) and *c*_2_(*k*) represent the error state dynamics projected on the subspace (unstable manifold) spanned by the basis vectors associated with the unstable FMs (*λ*_1_ and 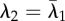), and *c*_3_(*k*) represents those on the subspace (stable manifold) spanned by the basis vectors associated with the stable FM (*λ*_3_). Red circles superposed on the waveforms represent stroboscopic observations of the coefficients with respect to the basis vectors. Vertical grey bars represent the double support phases and GRFs (N).

Figure 6 represents the stable and unstable gait dynamics shown in figure 2 in terms of the error state 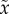. (Figure 5 shows the dominant eigenvectors as the function of *ϕ*_0_ for this unstable case.) One can confirm that all of three coefficients (*c*_1_, *c*_2_ and *c*_3_) that are observed stroboscopically at every *T* seconds asymptote to zero for the stable case, whereas *c*_1_ and *c*_2_ representing the two-dimensional unstable oscillatory dynamics associated with the two unstable FMs (*λ*_1_ and 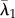) grow away from zero as described by equation (4.6) for the unstable case. Note that *c*_3_, which describes dynamics of the stable mode of the unstable dynamics, also diverges but slowly. That is, *c*_3_ evolves initially according to the converging dynamics of equation (4.7) when 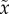 is small and the state point is close to the LC. However, as the state point becomes away from the LC due to the unstable dynamics of *c*_1_ and *c*_2_, the linear approximation of dynamics of the model becomes invalid, and thus *c*_3_ starts to move also away from the LC.

**Figure 7.**
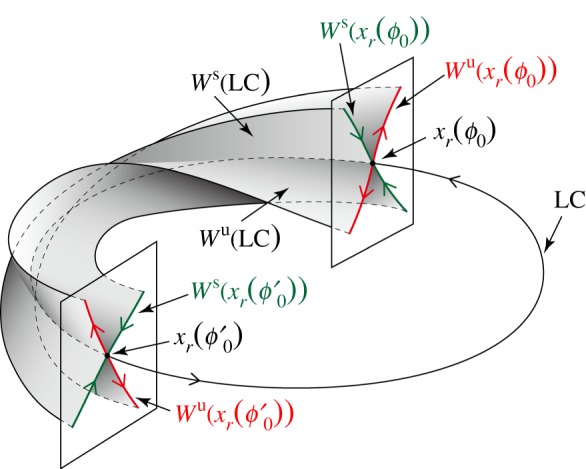
Schematic of stable and unstable manifolds of the LC. Twisted geometry of the manifolds corresponds to the *ϕ*_0_-dependent eigenvectors of 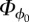. The stable manifold 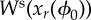 and the unstable manifold 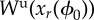 are, respectively, tangential to the subspace spanned by the eigenvectors for the stable FMs of 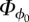 and that spanned by the set of eigenvectors for the unstable FMs of 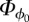. 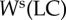 and 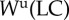 are the stable and unstable manifolds of the LC, respectively, and they are defined as the unions of 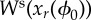 and 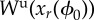 through the one gait cycle.

We shall make an association between the linear subspace spanned by eigenvectors of stable FMs and a stable manifold of the LC, and also between the linear subspace spanned by eigenvectors of unstable FMs and an unstable manifold of the LC. The stable manifold for any point 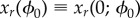 on the LC can be defined by the set of state points as
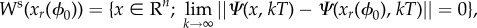
where *k* is integer representing the gait cycle, and 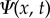 is the flow of the system, representing the evolution of the state point *x* for a time-span of *t*. Similarly, the unstable manifold for any point *x_r_*(*ϕ*_0_) on the LC is defined by the set of state points as
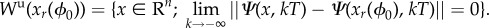
See figure 7 for a schematic of the stable and unstable manifolds of the LC.

The set 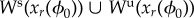 forms a Poincaré section of the LC passing through *x_r_*(*ϕ*_0_), across which any trajectory transverses periodically every *T* seconds. A Poincaré map for this section is defined for 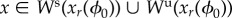 as
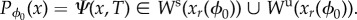


Moreover, since 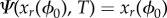,
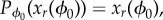
meaning that *x_r_*(*ϕ*_0_) is a fixed point of *Pϕ*_0_. If 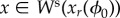,
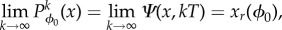
where iterative operations of 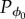 generate a convergent sequence of points on 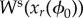 towards the fixed point *x_r_*(*ϕ*_0_) on the LC. Similarly, if 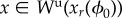,
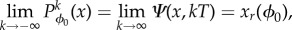
meaning that iterative operations of 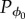 generate a divergent sequence of points on 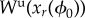 away from the fixed point 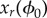 on the LC.

If the LC is stable, 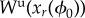 is an empty set, and there exists a non-empty set 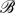 such that any state point in 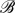 asymptotes to the LC. 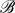 is called the basin of attraction of the LC defined as
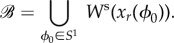


If the LC is unstable, it is typically accompanied by both 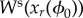 and 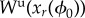, meaning that the fixed point 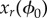 is a *saddle point*.

Since the matrix 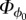 is a linearization of 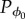 around the fixed point 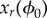, 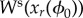 and 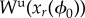 are, respectively, locally diffeomorphic with the subspace spanned by the set of eigenvectors for the stable FMs of 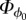 and that spanned by the set of eigenvectors for the unstable FMs of 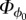. In other words, in the vicinity of the LC, 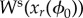 is identical with the linear subspace spanned by eigenvectors of stable FMs for 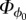, and also 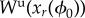 is identical with the linear subspace spanned by eigenvectors of unstable FMs for 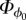. In the example shown in figure 6 for the unstable LC, the two-dimensional subspace spanned by 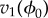 and 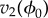 is locally identical with 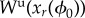, and the remaining 15-dimensional subspace, except the one-dimensional neutrally stable subspace, is locally identical with 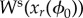.

In summary, we explored how stability of the model with the PD-feedback controller changes as the function of PD-gains that determine gait flexibility. Moreover, we characterized dynamics of the perturbed state point (error state point) in the vicinity of the LC using the stable and unstable manifolds of the LC. In particular, phase-dependent geometry of the stable and unstable manifolds was described by the eigenvectors of the monodromy matrix, by which we can understand that a perturbed state point at a given phase converges to the LC transiently along the stable manifold and diverges from the LC along the unstable manifold.

## 5. Stabilization of unstable gait using intermittent control

We have shown so far that the time-continuous PD-feedback controller can achieve stable gait if the PD-gains are large enough, which means that gait dynamics established by impedance control are not flexible but rigid. In this section, we propose a time-discontinuous, intermittent control strategy that can stabilize unstable dynamics of the biped gait model with small values of PD-gains, as an alternative to impedance control. More specifically, we show that the unstable dynamics of the biped model with small PD-gains can be stabilized by introducing an additional feedback controller, referred to as *the intermittent controller* that acts impulsively only at a specific optimal phase of every stride for a short period of time, referred to as the *on-period*. This means that the intermittent controller is *inactivated* for most of time, referred to as the *off-period*, during which the joints of the model are actuated only by the continuous PD-feedback controller with small gains, leading to a flexible gait with the overall low joint impedance.

### 5.1. Intermittent control using the stable manifold

The proposed intermittent controller is implemented also by a PD feedback controller. However, it is characterized differently by the fact that a desired state point at time *t* for this feedback controller is not the desired state *x_r_*(*t*; *ϕ*_0_) on the LC, but a nominal desired state point on the stable manifold 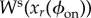, and the controller is activated at a specific phase *ϕ*_on_, referred to as *the onset phase* for a short duration *w*. It exploits the fact that a state point exactly on the stable manifold 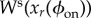 approaches the LC (the origin in terms of the error state 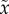) through repeated cycles even for the unstable gait. More specifically, 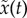 on 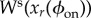 will be mapped to 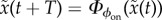 on 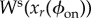 after one gait cycle, by which 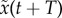 becomes closer to the origin than 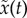. Therefore, if the state point can be forced to be close to the stable manifold by the intermittent controller in each on-period, it is expected that, after the following off-period of the intermittent controller, the state point at the next onset of the on-period may tend to approach the LC, at least for several repeated gait cycles, even if the state point is not exactly on the stable manifold.

There is a freedom of choice for the nominal desired state point, referred to as *x_s_*, on the stable manifold 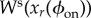. One can consider an optimal criterion to determine the nominal desired state point. However, in this study, we simply define it as the projection of the current state point *x*(*t*) onto the stable manifold as *x_s_*. Let us consider the intermittent controller that is activated at time *t* with an onset phase *ϕ*_on_. Since we assume a short duration *w* of the on-period (impulsive activation), we approximate the error state point 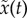 for the short time interval [*t*, *t* + *w*] in terms of the time-independent basis vectors at the onset phase *ϕ*_on_, which is expressed as5.1
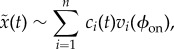
where *n* = 18, 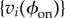 is the time-independent set of *n*-normalized eigenvectors of 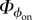, and *c_i_*(*t*) is the time-dependent coefficient for the *i*th time-independent basis vector 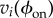.

Let us consider the case with two unstable FMs associated with 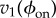 and 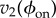 and the remaining stable FMs, except one unity FM, as in figures 5 and 6, right. In this case, the stable manifold 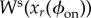 is locally spanned by the (*n* − 3) eigenvectors 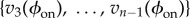, where the last *n*th eigenvector is reserved for the unity eigenvalue that is associated with the linear increasing in the position *x* of HAT-CoM. Thus, the projection of the current error state point on the stable manifold, i.e. the nominal desired state point 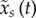 can be obtained as5.2
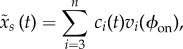
by putting the coefficients of the unstable modes to zeros, i.e. *c*_1_(*t*) = *c*_2_(*t*) = 0. Thus, the nominal state point in terms of the original coordinate is expressed as5.3
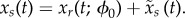
We then define *q_s_*(*t*), the nominal desired posture in terms of the generalized coordinate, as5.4
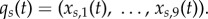


The intermittent controller is then implemented as follows:


5.5
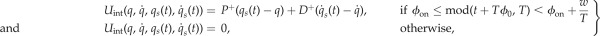


with *P* ^+^ and *D*^+^ being the proportional and derivative gain matrices defined as3.4
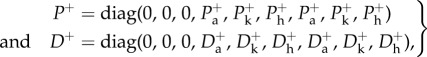
where 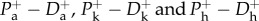 are the gains of intermittent PD feedback control acting on the ankles, knees and hips of the left and right limbs, respectively. We apply *U*_int_ to the right-hand-side of equation (2.4) intermittently at a certain onset phase *ϕ*_on_ only for *w* seconds. By taking into account the periodic nature and the left–right symmetry of the gait, we apply the intermittent control every half cycle, i.e. once for each step. In other words, each on-period of *U*_int_ begins when the phase of the desired trajectory 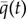 is at *ϕ*_on_, and then suspended to zero when the phase of the desired trajectory 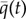 is at *ϕ*_on_ + *w*/*T* where the off-period begins. The subsequent on-period starts at *ϕ*_on_ + 0.5, and then suspended to zero at 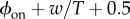. (Note that the small duration *w* is a parameter that determines the width of each on-period, which can be optimized for better stability as we show later in this section.) This means that the sequence of short-time torques (*U*_int_) is completely periodic, regardless of the state of the biped model. However, since we consider no-modifications of the desired trajectory, this condition for every onset of *U*_int_ based only on the phase can be considered as a simplification of a state-dependent activation of the intermittent controller. On the other hand, the condition for every offset is rather automatic. Therefore, although *U*_int_ drives the state point towards the nominal desired state point *x_s_* on the stable manifold 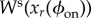, the state point at each offset of *U*_int_ (i.e. at the beginning of each off-period) is not necessarily close enough to the stable manifold 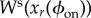.

**Figure 8.**
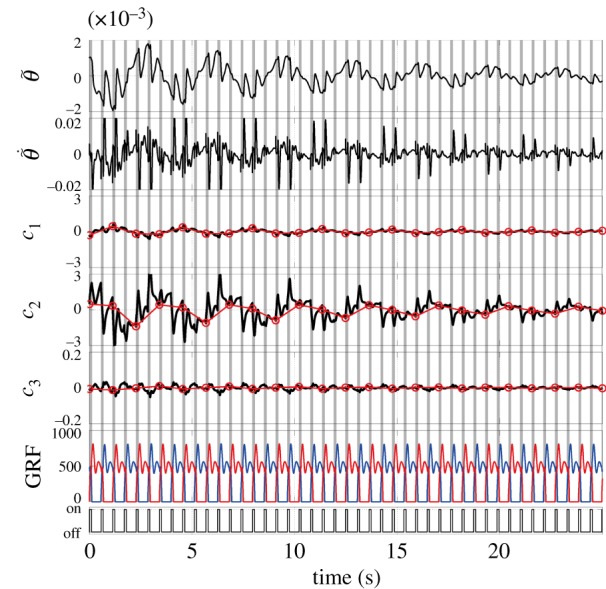
Successful stabilization of the unstable gait by the intermittent controller that uses the stable manifold of the unstable LC (SMC), i.e. the intermittent controller drives the state point toward the nominal desired state point on the stable manifold of the LC for a short period of time (*w* seconds) in every half cycle of gait. (*P*_a_, *P*_k_, *P*_h_) = (700, 700, 700) as in figure 6*b*, 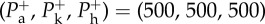 and 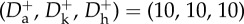. The onset phase was *ϕ*_on_ = 0.003, which is right after the heel-contact event, and the duration *w* was 0.10 s. Note that the sequence of on-periods at the bottom trace coincides with the vertical grey bands that represent the double support phases. 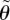 and 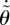: the error states of the tilt angle (rad) and angular velocity (rad s^–1^) of HAT. *c*_1_, *c*_2_, *c*_3_: the coefficients of three dominant basis vectors. GRF, ground reaction force (N).

Figure 8 exemplifies dynamics of the model with the intermittent controller *U*_int_, in which instability of the model due to small values of *P*-gains can be successfully compensated by the intermittent controller. In this case, the intermittent control torque is periodically activated at every double support phase at which the stance leg exchanges. One can compare figure 8 with figure 6, right, both of which are simulated for the identical initial condition and the small PD-gains. However, the model without the intermittent controller starts to fall rapidly in a few steps from the beginning of the simulation.

### 5.2. Intermittent control driving directly to the limit cycle

A question arises if the intermittent controller *U*_int_ is better to drive the state point to the stable manifold of the unstable LC. What will happen if the state point is not driven to the nominal desired state point on the stable manifold as examined above, but to the desired state point on the unstable LC directly as in the original PD-feedback controller *U*_fb_? It is natural to ask whether it is more convenient to use the stable manifold or ignore it. In order to answer this question, we consider another intermittent controller whose desired state point is 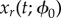 on the unstable LC.

If we use the state point 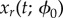 on the unstable LC as the desired state point for the intermittent controller, instead of the nominal desired state point on the stable manifold, the total feedback control torque during each on-period can be expressed as follows:5.6
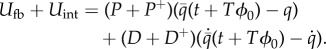
This is equivalent to putting 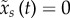 in equation (5.3) and replacing *q_s_*(*t*) and 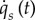 in equation (2.4) by 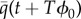 and 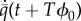.

**Figure 9.**
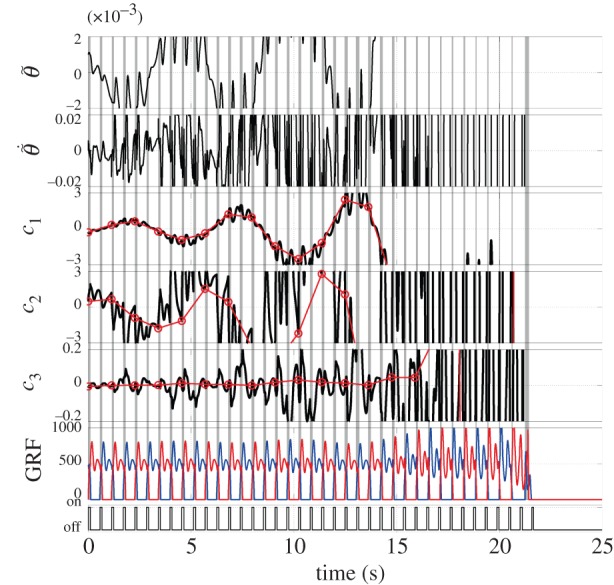
Failure in stabilizing the unstable gait by the intermittent controller that drives the state point directly to the desired state point on the unstable LC (LCC). The *P*-gains, *P*^+^, *D*^+^, the onset phase and the duration were all the same as figure 8.

Figure 9 exemplifies the dynamics of the model with the intermittent controller that drives the state point to the desired state point on the unstable LC directly, where the onset phase *ϕ*_on_, the activation duration *w* and the gains of the intermittent controller are the same as those used in figure 8. Despite this, however, instability due to small values of *P*-gains cannot be compensated by the intermittent controller that drives the state point directly back to the LC. This result suggests a superiority of the use the stable manifold of the unstable LC.

### 5.3. Comparison between two types of intermittent controllers

We examined the performance of two types of intermittent controllers in detail to determine which controller can better stabilize the unstable gait dynamics in a robust way. For convenience, we name the intermittent controller that drives the state point to the stable manifold as SMC (Stable Manifold Controller), and the other one that drives the state point directly to the unstable LC as LCC (Limit Cycle Controller).

**Figure 10.**
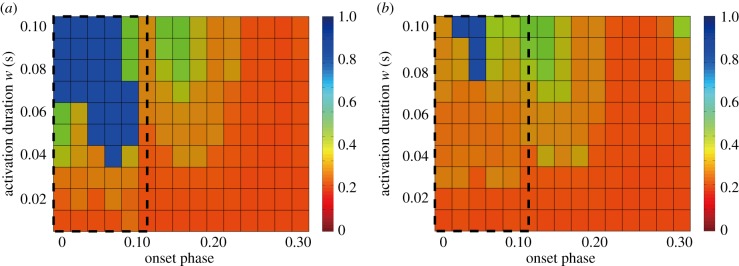
Optimal onset phase *ϕ*_on_ and duration *w* (s) of the intermittent activation for SMC (*a*) and LCC (*b*). For each set of *ϕ*_on_ and *w*, a simulation was performed for 100 cycles, and the cycle number *N*_fall_ when the model falls was recorded. Performance of SMC and LCC was evaluated by the ratio of *N*_fall_/100. Each grid of *ϕ*_on_−*w* plane is coloured by blue if the ratio is close to 1.0, and by red if it is close to zero, as indicated by the colour bars. In each panel, the intermittent control successfully stabilizes the unstable gait in the blue region, which is much wider for SMC than for LCC. (*P*_a_, *P*_k_, *P*_h_) = (700, 700, 700) as in figure 7. 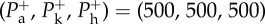 and 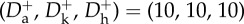 as in figures 8 and 9. Dotted square in each panel indicates the double support phase.

Since dynamics of the model with the intermittent control may depend on the onset phase *ϕ*_on_ and the activation duration *w*, we explored the optimal onset phase and the minimum duration for each type of the intermittent controller (figure 10). Note that the shorter the activation duration, the lower is the overall joint impedance. A set of numerical simulations showed that, for the stabilization of the unstable LC, *ϕ*_on_ should be located within the double support phase for both SMC and LCC, and the minimum duration *w* of each single activation is about 3.5% of the gait cycle for SMC, and about 7% for LCC. Since the duration of one of two double support phases in one gait cycle during steady-state walking in our model is about 10%, the duration *w* for SMC and LCC can be shorter than the double support phase.

**Figure 11.**
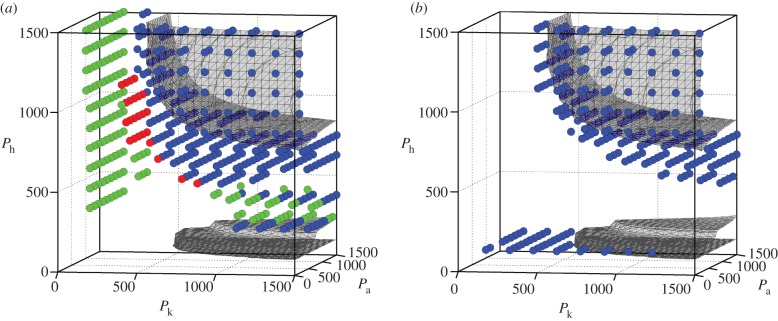
Stabilized regions in the *P*_a_*−P*_k_*−P*_h_ space by SMC (*a*) and LCC (*b*). Each coloured circle indicates that the unstable gait is stabilized by SMC in (*a*) and by LCC in (*b*). Colours of the circles represent how much the steady-state periodic solution *x*(*t*) of the model with SMC or LCC is distorted from the LC, which is evaluated by 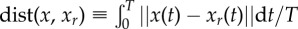. Blue circles indicate that dist(*x*, *x_r_*) is smaller than 0.05, which means that the solution of the model with SMC or LCC is close to the LC. Green circles indicate that dist(*x*, *x_r_*) is larger than 0.05 and smaller than 0.1. Red circles indicate that dist(*x*, *x_r_*) is larger than 0.1. (*P*_a_, *P*_k_, *P*_h_) = (700, 700, 700) as in figure 7. 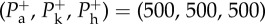 and 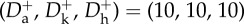 as in figures 8 and 9.

We also explored the performance of SMC and LCC (figure 11) for various sets of PD-gains for which the gait was determined as unstable as in figure 3 in the *P*_a_*−P*_k_*−P*_h_ parameter space, by examining whether SMC and LCC can successfully stabilize the unstable gait. It is apparent that SMC stabilizes the unstable gait for a wider range of the *P*_a_*−P*_k_*−P*_h_ parameter space than LCC. More specifically, LCC stabilizes the unstable gait only for the parameter regions in the neighbourhood of the original stability regions of the model without the intermittent control. Contrastingly, SMC stabilizes unstable gait even for parameter sets that are located far away from the original stability regions, implying robust stabilization capability of SMC.

In order to get more insights of how the unstable LC was stabilized by SMC and LCC, we examined the stabilized trajectories of the model in the state space, and evaluated how they were close to the LC. For both SMC and LCC, the stabilized trajectories were close to the LC when the set of *P*_a_*−P*_k_*−P*_h_ parameters were close to the original stability regions. However, the stabilized trajectories in SMC for the set of *P*_a_*−P*_k_*−P*_h_ parameters that are distant from the original stability regions were distorted from the LC. This is natural because the degree of instability becomes larger as the *P*-gains are far away from the instability point, and thus the impulsive feedback torque supplied by the SMC cannot drive the state point close enough to the stable manifold. However, driving the state point to the stable manifold is much easier than driving the state point directly to the LC, leading to the robust stabilization capability of SMC.

## 6. Discussion

In this paper, we addressed issues about gait stability during steady-state periodic walking with an anatomically plausible heel-toe footed biped model and investigated to which extent the human locomotion control system can achieve two apparently contrasting goals at the same time, namely dynamic stability and flexibility of gait. For example, one tolerates motor variability and the other does not [10]. In the field of neurophysiology, it has been a common view that the brain stabilizes unstable body dynamics using impedance control, which resists destabilizing motion by regulating co-activation levels and thus co-contraction levels of antagonist muscles [20,21]. Burdet *et al.* [39] have shown that the central nervous system stabilizes unstable dynamics by learning optimal impedance, in which only selected pairs of antagonist muscles associated with the instability are co-activated in a preprogrammed manner. However, a high impedance strategy, implemented by continuous co-contractions of antagonist muscles, is energetically expensive since it requires high metabolic costs, and sometimes it makes the body dynamics too rigid, leading to a loss of flexibility in pathological movements [13]. For a persistent, basic action such as human upright standing and walking, control strategies that disregard energy costs are not appropriate and/or physiologically plausible. This study focuses on the stability/flexibility issue and shows that intermittent feedback control is a promising alternative strategy that might resolve the trade-off between flexibility and stability, as well as between stability and energetic cost, although evaluation of the energetic issue, *per se*, is beyond the scope of this paper.

### 6.1. Summary

We showed that the desired steady-state gait, which we prepared carefully using a preliminary computational task, could be established as a stable LC of the model for large PD-feedback gains. Stability of the LC was explored systematically in the wide range of *P*_a_*−P*_k_*−P*_h_ parameter space using FMs. It was natural that the main stability region was located at large values of *P*-gains. More specifically, the hip joint should be stiff (*P*_h_ > 750 Nm rad^−1^), but the ankle joint can be quite flexible (*P*_a_ ∼ 250 Nm rad^−1^), and the knee joint should be very stiff with the flexible ankle joint, but it can be medium otherwise. In comparison with the recent reports by Shamaei *et al.* [22] on the quasi-stiffness during steady human gait, *P*_a_-values obtained in this study for the gait stability are roughly in the reported range (200–500 Nm rad^−1^). However, both *P*_k_-values and *P*_h_-values for the stability are far above the reported ranges (200–350 Nm rad^−1^ for knee [23] and 200–600 Nm rad^−1^ for hip [24]). Another thin horizontal stability region with *P*_h_ ∼ 100 Nm rad^−1^ for the hip joints could also provide stable gait as shown in figure 3, and this small value of *P*_h_ was close to the minimum value reported in [24]. However, the model predicted much larger stiffness for the knee and ankle joints in this thin stability region than the reported values. Thus, in any case, the model with the continuous PD-feedback controller could not achieve dynamic stability and flexibility simultaneously.

After we clarified the stable and unstable manifolds of the destabilized LC, we showed that the intermittent controller could better stabilize the unstable LC with small PD-gains, while leaving the overall joint impedance small by driving the state to the stable manifold of the unstable LC (SMC), not directly to the unstable LC itself (LCC). The comparison between SMC and LCC revealed a beneficial aspect of the use of the stable manifold of the saddle-type unstable LC dynamics. That is, SMC could stabilize the unstable LC for much wider region of the *P*-gains, suggesting a robustness of SMC. Examinations of our model showed an importance of the timing of active intervention during human gait. For the proposed intermittent control, we showed that the impulsive active control should be applied during the double support phase of the gait. This result is consistent with a well-known fact that the bipedal gait is close to uncontrollable during single support phase, but becomes controllable only during double support phase. Indeed, if there is no active ankle feedback torques, we can easily show that the body dynamics in terms of its angular momentum becomes completely uncontrollable.

### 6.2. Inverted pendulum analogy

The intermittent feedback torques, which are impulsively activated during the double support phase both in SMC and in LCC, might play roles to correct the joint torques for pushing forward (accelerating) and for pulling backward (decelerating) the inverted-pendulum-like body, resulting in, respectively, an enough initial velocity for the inverted pendulum to cross over the upright position during one single support phase and a termination of forward-falling of the inverted pendulum to prepare an appropriate initial velocity for the next single support phase. In this way, the proposed intermittent controller might have a high affinity for the inverted pendulum analogy of biped gait [40]. It is interesting to note that SMC and LCC might share the same functional roles in terms of the inverted pendulum analogy, but in different ways, and SMC could better achieve successive step-to-step transitions from one pendular stance to the next in a stable and robust manner than LCC, by using a well-timed neural intervention that exploits the stable modes (i.e. dynamics on the stable manifold) embedded in the unstable dynamics.

A future project of ours is to examine whether temporal patterns of the intermittent feedback torques (either with SMC or LCC) are consistent with the fly-ball analogy proposed by Kuo [40] as a dynamic version of the inverted pendulum analogy. Moreover, this examination should be performed together with the energetic comparison among different stabilization strategies, i.e. conventional impedance control, LCC and SMC.

### 6.3. Intermittent control as a hybrid dynamical system

Our gait model with the intermittent controller, both SMC and LCC, can be viewed as a hybrid dynamical system that switches discontinuously between two subsystems with and without the intermittent controller. SMC and LCC were used when the subsystem without the intermittent controller was unstable, that is, when the *P*-gains of the subsystem are outside the stability regions shown in figure 3. Here, we discuss stability of the LC for the subsystem with the intermittent controller if the intermittent controller is activated persistently, i.e. if *U*_fb_ + *U*_int_ defined by either equation (5.5) or equation (5.6) is used continuously.

In the case of LCC, the total *P*-gains of the feedback controller is *P* + *P*^+^. Thus, the LC of the subsystem with the continuous LCC is stable if the *P*-gains of the subsystem without LCC is located slightly below the main stability region of figure 3 and *P* + *P*^+^ is located inside the stability region. Indeed, the unstable LC was successfully stabilized mostly when the *P*-gains were distributed slightly below the main stability region of the subsystem without LCC. In this case, the hybrid dynamical system switches between the unstable subsystem without LCC and the stable subsystem with LCC, implying that dynamics of the stable subsystem with LCC during the on-period are responsible for the successful stabilization.

In the case of SMC, however, values of *P* + *P*^+^ are not necessarily located inside the stability region of the model without SMC, because the *P*-gains of the subsystem without SMC are not necessarily located in the neighbourhood of the main stability region shown in figure 3. This means that the hybrid dynamical system switches between the unstable subsystem without SMC and also the unstable subsystem with SMC. That is, the system alternates between two unstable dynamics, which makes overall dynamics stable. This is exactly the same situation in the intermittent postural control model during quiet standing [28,30,33].

### 6.4. Desired gait trajectory as a stability determinant

As we showed in equation (3.8), Floquet stability of the model with the continuous PD-feedback controller depends on the desired gait trajectory that was prescribed using a motion-captured joint angle trajectory and the constraint model. One may argue whether or not the optimally prescribed desired gait trajectory (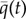 or 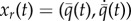), rather than the joint impedance and the feedback control strategies, is a key for gait stability, because we know intuitively that an apparently inappropriate desired gait trajectory can never be realized and stabilized by the model. Indeed, any desired gait trajectory *x_r_*(*t*) should be consistent at least with the zero moment point (ZMP) criterion [41], if we decide to use it for the model and wish to realize an actual gait motion of the model according to the desired gait trajectory. The ZMP criterion examines whether or not the prescribed desired gait trajectory *x_r_*(*t*) can be a solution of the equation of motion of the model (equation (2.4)), which is performed by substituting *x_r_*(*t*) into the equation of motion to calculate an acting point (ZMP) of unknown GRFs, and then examining whether the obtained ZMP is located inside the support area of feet: if not, otherwise, *x_r_*(*t*) cannot be physically realizable, and such *x_r_*(*t*) cannot be a solution of the equation of motion, regardless of control torques (*U*) applied to the joints. If a gait trajectory that does not satisfy the ZMP criterion is used as a desired gait trajectory to be tracked by the model, the model would fall inevitably. Thus, we can say that gait stability is most sensitive for a selection of the desired gait trajectory. However, the ZMP criterion is merely a necessary condition for gait stability. Even if the ZMP criterion is satisfied by *x_r_*(*t*) and if *x_r_*(*t*) is a solution of the equation of motion, *x_r_*(*t*) can be either stable or unstable. Indeed any gait dynamics of our model that is actuated only by the feed-forward torque *U*_ff_ could not be stable even if the *U*_ff_ is prepared for an optimally selected desired gait trajectory, implying the necessity of the feedback controller for gait stability.

One can also argue whether or not there exists a desired trajectory for performing human gait, including involvement of a central pattern generator (CPG) that might contribute to neural generation of the desired trajectory [42], emergence of gait patterns by means of purely mechanical mechanisms like in the passive gait [18] without using CPG or through bidirectional interactions between mechanical and neural dynamics of CPG [14,15]. Although, in reality, it is natural to assume that a basic gait pattern associated with a desired trajectory and joint impedance might be determined or optimized simultaneously somehow by the central nervous system, we have purposely avoided issues related to CPG and emergent property of a complex dynamical system, by which we could simply concentrate on the roles played by joint impedance and related neural control strategies. The results obtained by this study, however, might be applicable to a more complicated biped model that assumes a generation of the desired joint angle trajectory or the desired gait trajectory using a model of CPG.

### 6.5. Effects of feedback delay

Stability of the model with the continuous PD-feedback controller would alter if we consider a signal transmission delay of neural feedback control. Indeed, this has been one of the major concerns in the recent debate on how the human upright posture is stabilized in the presence of relatively large delay time that induces instability [27–30,32,33,43,44]. The stability regions obtained in this study for the biped model with the continuous PD-feedback controller (figure 3) might diminish largely if we consider feedback delay, in particular, for the large gain regime. This means that establishing gait stability by the conventional impedance control becomes much more difficult, if the continuous PD-feedback controller is implemented with delay.

Delay-induced instability in feedback control systems can be compensated by various control strategies. These include (i) acceleration feedback controller in addition to the PD-feedback controller [27], (ii) (intermittent) state predictor [44], (iii) (multiplicative) stochastic state feedback controller [45], as well as (iv) intermittent activation of the delayed-feedback controller [28,30,33], among others. Although the current study considered only the intermittent activation of the PD-feedback controller without feedback delay, even if a feedback delay is taken into account, we speculate that the intermittent feedback controller might still be able to show a good performance for compensating delay-induced instability and stabilizing unstable dynamics of the model with PD-gains far below the critical values, as in the case of quiet standing [28,30,33].

This speculation is supported by the following discussion. We first remind that the SMC was implemented by the intermittent PD-feedback controller that drives the state to the nominal desired state point on the stable manifold. We introduced the nominal desired state point and associated feedback controller for the sake of simplicity, as a matter of fact. The role played by this PD-controller *U*_int_ was to make the state point closer to the stable manifold during on-periods of its activation. *U*_int_ of LCC with feedback delay might be able to play a similar role more simply than SMC, without considering the nominal desired state point. In other words, if a neural feedback delay is taken into account for *U*_int_ of LCC, delay-induced unstable dynamics might be able to replace the role played by the current *U*_int_ of SMC that uses the nominal desired state point. This might be is achieved by the delay-induced unstable oscillatory dynamics around the destabilized fixed point in the system with LCC, which makes the state point get across the stable manifold without using the nominal desired state point, as in the intermittent postural controller [28,30,33].

### 6.6. Relation to phase resetting control

It is interesting to discuss complementary roles played by the phase resetting control [25,26] and the proposed intermittent controller. It has been shown that the phase resetting is effectively used by human subjects for maintaining gait when external perturbations are applied during swing phase, where typical motor responses depend on the phase of the perturbations [4]: perturbations applied at early or middle swing phase result in elevating strategy that is accompanied by phase delay of the gait cycle, whereas perturbations applied at late swing phase lead to lowering strategy that is accompanied by phase advance. However, it has been clarified that perturbations applied during double support phase do not elicit significant amount of phase resetting [26]. Contrastingly, the intermittent controller proposed in this study is effective during the double support phase. As Yamasaki *et al.* [25] has discussed, the phase resetting also contributes to reducing the joint impedance while maintaining gait stability. Together with the fact the proposed intermittent controller does not include any mechanisms for phase resetting, we can conclude that these two stabilization mechanisms are complementary, and effective to achieve gait stability with the lowest possible joint impedance.

### 6.7. Conclusion and future issues

In this study, we successfully extended the intermittent control paradigm, developed for quiet standing with the upright equilibrium state as a saddle-type fixed point, to steady-state human gait. This generalization was not trivial at all, since the gait trajectory is not a fixed point but rather a periodic motion modelled as an LC, while the concept of a saddle-like dynamics around the LC is still maintained. For stabilizing the unstable LC gait dynamics, we developed an intermittent neural feedback controller that is activated only for a short period of time at an optimal phase of each gait step. We characterized the robustness of this design by showing that it can better stabilize unstable gait with small continuous feedback gains, leading to a flexible gait. In particular, we demonstrated that such intermittent controller performs better if it drives the state point to the stable manifold of the unstable LC, rather than directly to the LC.

As our future issues, we are planning to analyse effects of noise and feedback delay with the gait model that involves both the intermittent controller and the phase resetting controller as the major stabilization mechanisms. Such study will allow us to examine motor variability in the model using the methodologies that have been developed for human gait data [3,8–10]. Since it has been shown that several types of intermittent control model during quiet standing could reproduce major characteristics of postural sway such as the power-law fluctuation [46], we expect that noisy dynamics of the intermittent gait control model might be able to provide possible mechanisms of how the physiological gait variability is generated.

## Supplementary Material

Equations of motion of the model and definition of joint impedance
