# Supplementary material for: An intermittent control model of flexible human gait using a stable manifold of saddle-type unstable limit cycle dynamics
Source: J R Soc Interface. 2014 Dec 6;11(101):20140958. doi: 10.1098/rsif.2014.0958 (PMC4223921; doi:10.1098/rsif.2014.0958)
Supplement: Equations of motion of the model and definition of joint impedance [file rsif20140958supp1.pdf]

Supplemental Materials

prepared for "An intermittent control model of flexible human gait using a stable manifold of saddle-type unstable limit cycle dynamics"

Chunjiang Fu[1], Yasuyuki Suzuki[1], Ken Kiyono[1], Pietro Morasso[2], Taishin Nomura[1]
1. Graduate School of Engineering Science, Osaka University, Toyonaka, Osaka, Japan
2. Italian Institute of Technology, Genoa, Italy

A Details of the model

A.1 Equations of motion

The equation of motion (Eqs. 4 and 5 in the main text) is rewritten as follows:

$$J\ddot{q}+B+K+G=U_{\mathrm{ff}}+U_{\mathrm{fb}}.$$

Elements of each of J, B, K, G, U_{ff} and U_{fb} are detailed here as follows:

$$
\begin{aligned}
J &= \begin{pmatrix} j_{1,1} & j_{1,2} & \cdots & j_{1,9} \\ j_{2,1} & j_{2,2} & & j_{2,9} \\ \vdots & & \ddots & \vdots \\ j_{9,1} & j_{9,2} & \cdots & j_{9,9} \end{pmatrix}, \\
B &= \begin{pmatrix} b_1 \\ b_2 \\ \vdots \\ b_9 \end{pmatrix}, \quad K = \begin{pmatrix} k_1 \\ k_2 \\ \vdots \\ k_9 \end{pmatrix}, \quad G = \begin{pmatrix} g_1 \\ g_2 \\ \vdots \\ g_9 \end{pmatrix}, \\
U_{\mathrm{ff}} &= \begin{pmatrix} 0 & 0 & 0 & u^l_{\mathrm{ff},a} & u^l_{\mathrm{ff},k} & u^l_{\mathrm{ff},h} & u^r_{\mathrm{ff},a} & u^r_{\mathrm{ff},k} & u^r_{\mathrm{ff},h} \end{pmatrix}^{\mathrm{T}}, \\
U_{\mathrm{fb}} &= \begin{pmatrix} 0 & 0 & 0 & u^l_{\mathrm{fb},a} & u^l_{\mathrm{fb},k} & u^l_{\mathrm{fb},h} & u^r_{\mathrm{fb},a} & u^r_{\mathrm{fb},k} & u^r_{\mathrm{fb},h} \end{pmatrix}^{\mathrm{T}} \\
j_{1,1} &= 2\left(I_1+m_1d_1^2\right)+2\left(I_2+m_1L_2^2+m_2d_2^2\right)+2\left(I_3+m_1L_3^2+m_2L_3^2+m_3d_3^2\right) \\
&\quad +\left(I_4+2(m_1+m_2+m_3)(d_4-L_4)^2\right) \\
&\quad +2m_1d_1\left\{L_2\cos\left(-\theta^l_a\right)+L_3\cos\left(-\theta^l_k-\theta^l_a\right)-(d_4-L_4)\cos\left(-\theta^l_h-\theta^l_k-\theta^l_a\right)\right\} \\
&\quad +2(m_1L_2+m_2d_2)\left\{L_3\cos\left(-\theta^l_k\right)-(d_4-L_4)\cos\left(-\theta^l_h-\theta^l_k\right)\right\} \\
&\quad -2(m_1L_3+m_2L_3+m_3d_3)(d_4-L_4)\cos\left(-\theta^l_h\right) \\
&\quad +2m_1d_1\{L_2\cos(-\theta^r_a)+L_3\cos(-\theta^r_k-\theta^r_a)-(d_4-L_4)\cos(-\theta^r_h-\theta^r_k-\theta^r_a)\} \\
&\quad +2(m_1L_2+m_2d_2)\{L_3\cos(-\theta^r_k)-(d_4-L_4)\cos(-\theta^r_h-\theta^r_k)\} \\
&\quad -2(m_1L_3+m_2L_3+m_3d_3)(d_4-L_4)\cos(-\theta^r_h) \\
j_{1,2} &= 2(m_1+m_2+m_3)(d_4-L_4)\sin(\theta) \\
&\quad -m_1d_1\sin\left(\theta-\theta^l_h-\theta^l_k-\theta^l_a\right) \\
&\quad -(m_1L_2+m_2d_2)\sin\left(\theta-\theta^l_h-\theta^l_k\right)
\end{aligned}
$$

$$
\begin{aligned}
& -(m_1L_3+m_2L_3+m_3d_3)\sin\left(\theta-\theta_h^l\right) \\
& -m_1d_1\sin(\theta-\theta_h^r-\theta_k^r-\theta_a^r) \\
& -(m_1L_2+m_2d_2)\sin(\theta-\theta_h^r-\theta_k^r) \\
& -(m_1L_3+m_2L_3+m_3d_3)\sin(\theta-\theta_h^r) \\
j_{1,3} \quad = \quad & -2(m_1+m_2+m_3)(d_4-L_4)\cos(\theta) \\
& +m_1d_1\cos\left(\theta-\theta_h^l-\theta_k^l-\theta_a^l\right) \\
& +(m_1L_2+m_2d_2)\cos\left(\theta-\theta_h^l-\theta_k^l\right) \\
& +(m_1L_3+m_2L_3+m_3d_3)\cos\left(\theta-\theta_h^l\right) \\
& +m_1d_1\cos(\theta-\theta_h^r-\theta_k^r-\theta_a^r) \\
& +(m_1L_2+m_2d_2)\cos(\theta-\theta_h^r-\theta_k^r) \\
& +(m_1L_3+m_2L_3+m_3d_3)\cos(\theta-\theta_h^r) \\
j_{1,4} \quad = \quad & -\left(I_1+m_1d_1^2\right) \\
& -m_1d_1L_2\cos\left(-\theta_a^l\right) \\
& -m_1d_1L_3\cos\left(-\theta_k^l-\theta_a^l\right) \\
& +m_1d_1(d_4-L_4)\cos\left(-\theta_h^l-\theta_k^l-\theta_a^l\right) \\
j_{1,5} \quad = \quad & -\left(I_1+m_1d_1^2\right)-\left(I_2+m_1L_2^2+m_2d_2^2\right) \\
& -2m_1d_1L_2\cos\left(-\theta_a^l\right) \\
& -m_1d_1L_3\cos\left(-\theta_k^l-\theta_a^l\right) \\
& +m_1d_1(d_4-L_4)\cos\left(-\theta_h^l-\theta_k^l-\theta_a^l\right) \\
& -(m_1L_2+m_2d_2)L_3\cos\left(-\theta_k^l\right) \\
& +(m_1L_2+m_2d_2)(d_4-L_4)\cos\left(-\theta_h^l-\theta_k^l\right) \\
j_{1,6} \quad = \quad & -\left(I_1+m_1d_1^2\right)-\left(I_2+m_1L_2^2+m_2d_2^2\right)-\left(I_3+m_1L_3^2+m_2L_3^2+m_3d_3^2\right) \\
& -2m_1d_1L_2\cos\left(-\theta_a^l\right) \\
& -2m_1d_1L_3\cos\left(-\theta_k^l-\theta_a^l\right) \\
& +m_1d_1(d_4-L_4)\cos\left(-\theta_h^l-\theta_k^l-\theta_a^l\right) \\
& -2(m_1L_2+m_2d_2)L_3\cos\left(-\theta_k^l\right) \\
& +(m_1L_2+m_2d_2)(d_4-L_4)\cos\left(-\theta_h^l-\theta_k^l\right) \\
& +(m_1L_3+m_2L_3+m_3d_3)(d_4-L_4)\cos\left(-\theta_h^l\right) \\
j_{1,7} \quad = \quad & -\left(I_1+m_1d_1^2\right) \\
& -m_1d_1L_2\cos(-\theta_a^r) \\
& -m_1d_1L_3\cos(-\theta_k^r-\theta_a^r)
\end{aligned}
$$

$$
\begin{aligned}
& +m_1d_1(d_4-L_4)\cos(-\theta_h^r-\theta_k^r-\theta_a^r) \\
j_{1,8} \;=\; & -\big(I_1+m_1d_1^2\big)-\big(I_2+m_1L_2^2+m_2d_2^2\big) \\
& -2m_1d_1L_2\cos(-\theta_a^r) \\
& -m_1d_1L_3\cos(-\theta_k^r-\theta_a^r) \\
& +m_1d_1(d_4-L_4)\cos(-\theta_h^r-\theta_k^r-\theta_a^r) \\
& -(m_1L_2+m_2d_2)L_3\cos(-\theta_k^r) \\
& +(m_1L_2+m_2d_2)(d_4-L_4)\cos(-\theta_h^r-\theta_k^r) \\
j_{1,9} \;=\; & -\big(I_1+m_1d_1^2\big)-\big(I_2+m_1L_2^2+m_2d_2^2\big)-\big(I_3+m_1L_3^2+m_2L_3^2+m_3d_3^2\big) \\
& -2m_1d_1L_2\cos(-\theta_a^r) \\
& -2m_1d_1L_3\cos(-\theta_k^r-\theta_a^r) \\
& +m_1d_1(d_4-L_4)\cos(-\theta_h^r-\theta_k^r-\theta_a^r) \\
& -2(m_1L_2+m_2d_2)L_3\cos(-\theta_k^r) \\
& +(m_1L_2+m_2d_2)(d_4-L_4)\cos(-\theta_h^r-\theta_k^r) \\
& +(m_1L_3+m_2L_3+m_3d_3)(d_4-L_4)\cos(-\theta_h^r) \\
j_{2,1} \;=\; & 2(m_1+m_2+m_3)(d_4-L_4)\sin(\theta) \\
& -m_1d_1\sin\Big(\theta-\theta_h^l-\theta_k^l-\theta_a^l\Big) \\
& -(m_1L_2+m_2d_2)\sin\Big(\theta-\theta_h^l-\theta_k^l\Big) \\
& -(m_1L_3+m_2L_3+m_3d_3)\sin\Big(\theta-\theta_h^l\Big) \\
& -m_1d_1\sin(\theta-\theta_h^r-\theta_k^r-\theta_a^r) \\
& -(m_1L_2+m_2d_2)\sin(\theta-\theta_h^r-\theta_k^r) \\
& -(m_1L_3+m_2L_3+m_3d_3)\sin(\theta-\theta_h^r) \\
j_{2,2} \;=\; & 2(m_1+m_2+m_3)+m_4 \\
j_{2,3} \;=\; & 0 \\
j_{2,4} \;=\; & m_1d_1\sin\Big(\theta-\theta_h^l-\theta_k^l-\theta_a^l\Big) \\
j_{2,5} \;=\; & m_1d_1\sin\Big(\theta-\theta_h^l-\theta_k^l-\theta_a^l\Big) \\
& +(m_1L_2+m_2d_2)\sin\Big(\theta-\theta_h^l-\theta_k^l\Big) \\
j_{2,6} \;=\; & m_1d_1\sin\Big(\theta-\theta_h^l-\theta_k^l-\theta_a^l\Big) \\
& +(m_1L_2+m_2d_2)\sin\Big(\theta-\theta_h^l-\theta_k^l\Big) \\
& +(m_1L_3+m_2L_3+m_3d_3)\sin\Big(\theta-\theta_h^l\Big) \\
j_{2,7} \;=\; & m_1d_1\sin(\theta-\theta_h^r-\theta_k^r-\theta_a^r) \\
j_{2,8} \;=\; & m_1d_1\sin(\theta-\theta_h^r-\theta_k^r-\theta_a^r) \\
& +(m_1L_2+m_2d_2)\sin(\theta-\theta_h^r-\theta_k^r) \\
j_{2,9} \;=\; & m_1d_1\sin(\theta-\theta_h^r-\theta_k^r-\theta_a^r) \\
& +(m_1L_2+m_2d_2)\sin(\theta-\theta_h^r-\theta_k^r) \\
& +(m_1L_3+m_2L_3+m_3d_3)\sin(\theta-\theta_h^r)
\end{aligned}
$$

$$
\begin{aligned}
j_{3,1} &= -2(m_1+m_2+m_3)(d_4-L_4)\cos(\theta) \\
&\quad +m_1 d_1 \cos\left(\theta-\theta_h^l-\theta_k^l-\theta_a^l\right) \\
&\quad +(m_1 L_2+m_2 d_2)\cos\left(\theta-\theta_h^l-\theta_k^l\right) \\
&\quad +(m_1 L_3+m_2 L_3+m_3 d_3)\cos\left(\theta-\theta_h^l\right) \\
&\quad +m_1 d_1 \cos(\theta-\theta_h^r-\theta_k^r-\theta_a^r) \\
&\quad +(m_1 L_2+m_2 d_2)\cos(\theta-\theta_h^r-\theta_k^r) \\
&\quad +(m_1 L_3+m_2 L_3+m_3 d_3)\cos(\theta-\theta_h^r) \\
j_{3,2} &= 0 \\
j_{3,3} &= 2(m_1+m_2+m_3)+m_4 \\
j_{3,4} &= -m_1 d_1 \cos\left(\theta-\theta_h^l-\theta_k^l-\theta_a^l\right) \\
j_{3,5} &= -m_1 d_1 \cos\left(\theta-\theta_h^l-\theta_k^l-\theta_a^l\right) \\
&\quad -(m_1 L_2+m_2 d_2)\cos\left(\theta-\theta_h^l-\theta_k^l\right) \\
j_{3,6} &= -m_1 d_1 \cos\left(\theta-\theta_h^l-\theta_k^l-\theta_a^l\right) \\
&\quad -(m_1 L_2+m_2 d_2)\cos\left(\theta-\theta_h^l-\theta_k^l\right) \\
&\quad -(m_1 L_3+m_2 L_3+m_3 d_3)\cos\left(\theta-\theta_h^l\right) \\
j_{3,7} &= -m_1 d_1 \cos(\theta-\theta_h^r-\theta_k^r-\theta_a^r) \\
j_{3,8} &= -m_1 d_1 \cos(\theta-\theta_h^r-\theta_k^r-\theta_a^r) \\
&\quad -(m_1 L_2+m_2 d_2)\cos(\theta-\theta_h^r-\theta_k^r) \\
j_{3,9} &= -m_1 d_1 \cos(\theta-\theta_h^r-\theta_k^r-\theta_a^r) \\
&\quad -(m_1 L_2+m_2 d_2)\cos(\theta-\theta_h^r-\theta_k^r) \\
&\quad -(m_1 L_3+m_2 L_3+m_3 d_3)\cos(\theta-\theta_h^r) \\
j_{4,1} &= -\left(I_1+m_1 d_1^2\right) \\
&\quad -m_1 d_1 L_2 \cos\left(-\theta_a^l\right) \\
&\quad -m_1 d_1 L_3 \cos\left(-\theta_k^l-\theta_a^l\right) \\
&\quad +m_1 d_1 (d_4-L_4)\cos\left(-\theta_h^l-\theta_k^l-\theta_a^l\right) \\
j_{4,2} &= m_1 d_1 \sin\left(\theta-\theta_h^l-\theta_k^l-\theta_a^l\right) \\
j_{4,3} &= -m_1 d_1 \cos\left(\theta-\theta_h^l-\theta_k^l-\theta_a^l\right) \\
j_{4,4} &= I_1+m_1 d_1^2 \\
j_{4,5} &= \left(I_1+m_1 d_1^2\right) \\
&\quad +m_1 d_1 L_2 \cos\left(-\theta_a^l\right) \\
j_{4,6} &= \left(I_1+m_1 d_1^2\right) \\
&\quad +m_1 d_1 L_2 \cos\left(-\theta_a^l\right)
\end{aligned}
$$

$$
\begin{aligned}
& +m_1 d_1 L_3 \cos\left(-\theta_k^l - \theta_a^l\right) \\
j_{4,7} &= 0 \\
j_{4,8} &= 0 \\
j_{4,9} &= 0 \\
j_{5,1} &= -\left(I_1 + m_1 d_1^2\right) - \left(I_2 + m_1 L_2^2 + m_2 d_2^2\right) \\
& -2 m_1 d_1 L_2 \cos\left(-\theta_a^l\right) \\
& -m_1 d_1 L_3 \cos\left(-\theta_k^l - \theta_a^l\right) \\
& +m_1 d_1 (d_4 - L_4) \cos\left(-\theta_h^l - \theta_k^l - \theta_a^l\right) \\
& -(m_1 L_2 + m_2 d_2) L_3 \cos\left(-\theta_k^l\right) \\
& +(m_1 L_2 + m_2 d_2)(d_4 - L_4) \cos\left(-\theta_h^l - \theta_k^l\right) \\
j_{5,2} &= (m_1 L_2 + m_2 d_2) \sin\left(\theta - \theta_h^l - \theta_k^l\right) \\
& +m_1 d_1 \sin\left(\theta - \theta_h^l - \theta_k^l - \theta_a^l\right) \\
j_{5,3} &= -(m_1 L_2 + m_2 d_2) \cos\left(\theta - \theta_h^l - \theta_k^l\right) \\
& -m_1 d_1 \cos\left(\theta - \theta_h^l - \theta_k^l - \theta_a^l\right) \\
j_{5,4} &= \left(I_1 + m_1 d_1^2\right) \\
& +m_1 d_1 L_2 \cos\left(-\theta_a^l\right) \\
j_{5,5} &= \left(I_1 + m_1 d_1^2\right) \\
& +2 m_1 d_1 L_2 \cos\left(-\theta_a^l\right) \\
& +\left(I_2 + m_1 L_2^2 + m_2 d_2^2\right) \\
j_{5,6} &= \left(I_1 + m_1 d_1^2\right) + \left(I_2 + m_1 L_2^2 + m_2 d_2^2\right) \\
& +2 m_1 d_1 L_2 \cos\left(-\theta_a^l\right) \\
& +m_1 d_1 L_3 \cos\left(-\theta_k^l - \theta_a^l\right) \\
& +(m_1 L_2 + m_2 d_2) L_3 \cos\left(-\theta_k^l\right) \\
j_{5,7} &= 0 \\
j_{5,8} &= 0 \\
j_{5,9} &= 0 \\
j_{6,1} &= -\left(I_1 + m_1 d_1^2\right) - \left(I_2 + m_1 L_2^2 + m_2 d_2^2\right) - \left(I_3 + m_1 L_3^2 + m_2 L_3^2 + m_3 d_3^2\right) \\
& -2 m_1 d_1 L_2 \cos\left(-\theta_a^l\right) \\
& -2 m_1 d_1 L_3 \cos\left(-\theta_k^l - \theta_a^l\right) \\
& +m_1 d_1 (d_4 - L_4) \cos\left(-\theta_h^l - \theta_k^l - \theta_a^l\right) \\
& -2(m_1 L_2 + m_2 d_2) L_3 \cos\left(-\theta_k^l\right)
\end{aligned}
$$

$$
\begin{aligned}
& +(m_1L_2+m_2d_2)(d_4-L_4)\cos\left(-\theta_h^l-\theta_k^l\right) \\
& +(m_1L_3+m_2L_3+m_3d_3)(d_4-L_4)\cos\left(-\theta_h^l\right) \\
j_{6,2} &= m_1d_1\sin\left(\theta-\theta_h^l-\theta_k^l-\theta_a^l\right) \\
& +(m_1L_2+m_2d_2)\sin\left(\theta-\theta_h^l-\theta_k^l\right) \\
& +(m_1L_3+m_2L_3+m_3d_3)\sin\left(\theta-\theta_h^l\right) \\
j_{6,3} &= -m_1d_1\cos\left(\theta-\theta_h^l-\theta_k^l-\theta_a^l\right) \\
& -(m_1L_2+m_2d_2)\cos\left(\theta-\theta_h^l-\theta_k^l\right) \\
& -(m_1L_3+m_2L_3+m_3d_3)\cos\left(\theta-\theta_h^l\right) \\
j_{6,4} &= \left(I_1+m_1d_1^2\right) \\
& +m_1d_1L_2\cos\left(-\theta_a^l\right) \\
& +m_1d_1L_3\cos\left(-\theta_k^l-\theta_a^l\right) \\
j_{6,5} &= \left(I_1+m_1d_1^2\right)+\left(I_2+m_1L_2^2+m_2d_2^2\right) \\
& +2m_1d_1L_2\cos\left(-\theta_a^l\right) \\
& +m_1d_1L_3\cos\left(-\theta_k^l-\theta_a^l\right) \\
& +(m_1L_2+m_2d_2)L_3\cos\left(-\theta_k^l\right) \\
j_{6,6} &= m_1d_1L_3\cos\left(-\theta_k^l-\theta_a^l\right)+(m_1L_2L_3+m_2d_2L_3)\cos\left(-\theta_k^l\right) \\
& +\left(I_3+m_1L_3^2+m_2L_3^2+m_3d_3^2\right)+\left(I_1+m_1d_1^2\right)+m_1d_1L_2\cos\left(-\theta_a^l\right) \\
& +m_1d_1L_3\cos\left(-\theta_k^l-\theta_a^l\right)+m_1d_1L_2\cos\left(-\theta_a^l\right)+\left(I_2+m_1L_2^2+m_2d_2^2\right) \\
& +(m_1L_2+m_2d_2)L_3\cos\left(-\theta_k^l\right) \\
j_{6,7} &= 0 \\
j_{6,8} &= 0 \\
j_{6,9} &= 0 \\
j_{7,1} &= -\left(I_1+m_1d_1^2\right) \\
& -m_1d_1L_2\cos(-\theta_a^r) \\
& -m_1d_1L_3\cos(-\theta_k^r-\theta_a^r) \\
& +m_1d_1(d_4-L_4)\cos(-\theta_h^r-\theta_k^r-\theta_a^r) \\
j_{7,2} &= m_1d_1\sin(\theta-\theta_h^r-\theta_k^r-\theta_a^r) \\
j_{7,3} &= -m_1d_1\cos(\theta-\theta_h^r-\theta_k^r-\theta_a^r) \\
j_{7,4} &= 0 \\
j_{7,5} &= 0 \\
j_{7,6} &= 0 \\
j_{7,7} &= I_1+m_1d_1^2
\end{aligned}
$$

$$
\begin{aligned}
j_{7,8} &= \left(I_1+m_1 d_1^2\right) \\
&\quad +m_1 d_1 L_2 \cos(-\theta_a^r) \\
j_{7,9} &= \left(I_1+m_1 d_1^2\right) \\
&\quad +m_1 d_1 L_2 \cos(-\theta_a^r) \\
&\quad +m_1 d_1 L_3 \cos(-\theta_k^r-\theta_a^r) \\
j_{8,1} &= (m_1 L_2+m_2 d_2)(d_4-L_4)\cos(-\theta_h^r-\theta_k^r) \\
&\quad -m_1 d_1 L_2 \cos(-\theta_a^r) \\
&\quad -\left(I_2+m_1 L_2^2+m_2 d_2^2\right) \\
&\quad -(m_1 L_2 L_3+m_2 d_2 L_3)\cos(-\theta_k^r) \\
&\quad +m_1 d_1 (d_4-L_4)\cos(-\theta_h^r-\theta_k^r-\theta_a^r) \\
&\quad -\left(I_1+m_1 d_1^2\right) \\
&\quad -m_1 d_1 L_2 \cos(-\theta_a^r) \\
&\quad -m_1 d_1 L_3 \cos(-\theta_k^r-\theta_a^r) \\
j_{8,2} &= (m_1 L_2+m_2 d_2)\sin(\theta-\theta_h^r-\theta_k^r) \\
&\quad +m_1 d_1 \sin(\theta-\theta_h^r-\theta_k^r-\theta_a^r) \\
j_{8,3} &= -(m_1 L_2+m_2 d_2)\cos(\theta-\theta_h^r-\theta_k^r) \\
&\quad -m_1 d_1 \cos(\theta-\theta_h^r-\theta_k^r-\theta_a^r) \\
j_{8,4} &= 0 \\
j_{8,5} &= 0 \\
j_{8,6} &= 0 \\
j_{8,7} &= \left(I_1+m_1 d_1^2\right) \\
&\quad +m_1 d_1 L_2 \cos(-\theta_a^r) \\
j_{8,8} &= \left(I_1+m_1 d_1^2\right)+\left(I_2+m_1 L_2^2+m_2 d_2^2\right) \\
&\quad +2 m_1 d_1 L_2 \cos(-\theta_a^r) \\
j_{8,9} &= \left(I_1+m_1 d_1^2\right)+\left(I_2+m_1 L_2^2+m_2 d_2^2\right) \\
&\quad +2 m_1 d_1 L_2 \cos(-\theta_a^r) \\
&\quad +m_1 d_1 L_3 \cos(-\theta_k^r-\theta_a^r) \\
&\quad +(m_1 L_2+m_2 d_2) L_3 \cos(-\theta_k^r) \\
j_{9,1} &= -\left(I_1+m_1 d_1^2\right)-\left(I_2+m_1 L_2^2+m_2 d_2^2\right)-\left(I_3+m_1 L_3^2+m_2 L_3^2+m_3 d_3^2\right) \\
&\quad -2 m_1 d_1 L_2 \cos(-\theta_a^r) \\
&\quad -2 m_1 d_1 L_3 \cos(-\theta_k^r-\theta_a^r) \\
&\quad +m_1 d_1 (d_4-L_4)\cos(-\theta_h^r-\theta_k^r-\theta_a^r) \\
&\quad -2(m_1 L_2+m_2 d_2) L_3 \cos(-\theta_k^r) \\
&\quad +(m_1 L_2+m_2 d_2)(d_4-L_4)\cos(-\theta_h^r-\theta_k^r) \\
&\quad +(m_1 L_3+m_2 L_3+m_3 d_3)(d_4-L_4)\cos(-\theta_h^r) \\
j_{9,2} &= (m_1 L_3+m_2 L_3+m_3 d_3)\sin(\theta-\theta_h^r) \\
&\quad +(m_1 L_2+m_2 d_2)\sin(\theta-\theta_h^r-\theta_k^r) \\
&\quad +m_1 d_1 \sin(\theta-\theta_h^r-\theta_k^r-\theta_a^r) \\
j_{9,3} &= -(m_1 L_3+m_2 L_3+m_3 d_3)\cos(\theta-\theta_h^r)
\end{aligned}
$$

$$
\begin{aligned}
& -(m_1L_2{+}m_2d_2)\cos(\theta{-}\theta_h^r{-}\theta_k^r) \\
& -m_1d_1\cos(\theta{-}\theta_h^r{-}\theta_k^r{-}\theta_a^r) \\
j_{9,4} &= 0 \\
j_{9,5} &= 0 \\
j_{9,6} &= 0 \\
j_{9,7} &= \left(I_1{+}m_1d_1^2\right) \\
& +m_1d_1L_2\cos(-\theta_a^r) \\
& +m_1d_1L_3\cos(-\theta_k^r{-}\theta_a^r) \\
j_{9,8} &= \left(I_1{+}m_1d_1^2\right){+}\left(I_2{+}m_1L_2^2{+}m_2d_2^2\right) \\
& +2m_1d_1L_2\cos(-\theta_a^r) \\
& +m_1d_1L_3\cos(-\theta_k^r{-}\theta_a^r) \\
& +(m_1L_2{+}m_2d_2)L_3\cos(-\theta_k^r) \\
j_{9,9} &= \left(I_1{+}m_1d_1^2\right){+}\left(I_2{+}m_1L_2^2{+}m_2d_2^2\right){+}\left(I_3{+}m_1L_3^2{+}m_2L_3^2{+}m_3d_3^2\right) \\
& +2m_1d_1L_2\cos(-\theta_a^r) \\
& +2m_1d_1L_3\cos(-\theta_k^r{-}\theta_a^r) \\
& +2(m_1L_2{+}m_2d_2)L_3\cos(-\theta_k^r) \\
b_1 &= -m_1d_1(d_4{-}L_4)\left\{\dot\theta^2{-}\left(\dot\theta{-}\dot\theta_h^l{-}\dot\theta_k^l{-}\dot\theta_a^l\right)^2\right\}\sin\left(-\theta_h^l{-}\theta_k^l{-}\theta_a^l\right) \\
& -m_1d_1(d_4{-}L_4)\left\{\dot\theta^2{-}\left(\dot\theta{-}\dot\theta_h^r{-}\dot\theta_k^r{-}\dot\theta_a^r\right)^2\right\}\sin(-\theta_h^r{-}\theta_k^r{-}\theta_a^r) \\
& -(m_1L_2{+}m_2d_2)(d_4{-}L_4)\left\{\dot\theta^2{-}\left(\dot\theta{-}\dot\theta_h^l{-}\dot\theta_k^l\right)^2\right\}\sin\left(-\theta_h^l{-}\theta_k^l\right) \\
& -(m_1L_2{+}m_2d_2)(d_4{-}L_4)\left\{\dot\theta^2{-}\left(\dot\theta{-}\dot\theta_h^r{-}\dot\theta_k^r\right)^2\right\}\sin(-\theta_h^r{-}\theta_k^r) \\
& -(m_1L_3{+}m_2L_3{+}m_3d_3)(d_4{-}L_4)\left\{\dot\theta^2{-}\left(\dot\theta{-}\dot\theta_h^l\right)^2\right\}\sin\left(-\theta_h^l\right) \\
& -(m_1L_3{+}m_2L_3{+}m_3d_3)(d_4{-}L_4)\left\{\dot\theta^2{-}\left(\dot\theta{-}\dot\theta_h^r\right)^2\right\}\sin(-\theta_h^r) \\
& -m_1d_1L_3\left\{\left(\dot\theta{-}\dot\theta_h^l{-}\dot\theta_k^l{-}\dot\theta_a^l\right)^2{-}\left(\dot\theta{-}\dot\theta_h^l\right)^2\right\}\sin\left(-\theta_k^l{-}\theta_a^l\right) \\
& -(m_1L_2{+}m_2d_2)L_3\left\{\left(\dot\theta{-}\dot\theta_h^l{-}\dot\theta_k^l\right)^2{-}\left(\dot\theta{-}\dot\theta_h^l\right)^2\right\}\sin\left(-\theta_k^l\right) \\
& -m_1d_1L_2\left\{\left(\dot\theta{-}\dot\theta_h^l{-}\dot\theta_k^l{-}\dot\theta_a^l\right)^2{-}\left(\dot\theta{-}\dot\theta_h^l{-}\dot\theta_k^l\right)^2\right\}\sin\left(-\theta_a^l\right) \\
& -m_1d_1L_3\left\{\left(\dot\theta{-}\dot\theta_h^r{-}\dot\theta_k^r{-}\dot\theta_a^r\right)^2{-}\left(\dot\theta{-}\dot\theta_h^r\right)^2\right\}\sin(-\theta_k^r{-}\theta_a^r) \\
& -(m_1L_2{+}m_2d_2)L_3\left\{\left(\dot\theta{-}\dot\theta_h^r{-}\dot\theta_k^r\right)^2{-}\left(\dot\theta{-}\dot\theta_h^r\right)^2\right\}\sin(-\theta_k^r) \\
& -m_1d_1L_2\left\{\left(\dot\theta{-}\dot\theta_h^r{-}\dot\theta_k^r{-}\dot\theta_a^r\right)^2{-}\left(\dot\theta{-}\dot\theta_h^r{-}\dot\theta_k^r\right)^2\right\}\sin(-\theta_a^r)
\end{aligned}
$$

$$
\begin{aligned}
b_2 \;=\;& -m_1 d_1 \left\{ \left(\dot\theta - \dot\theta_h^l - \dot\theta_k^l - \dot\theta_a^l\right)^2 \cos\left(\theta - \theta_h^l - \theta_k^l - \theta_a^l\right) + \left(\dot\theta - \dot\theta_h^r - \dot\theta_k^r - \dot\theta_a^r\right)^2 \cos(\theta - \theta_h^r - \theta_k^r - \theta_a^r) \right\} \\
& -(m_1 L_2 + m_2 d_2) \left\{ \left(\dot\theta - \dot\theta_h^l - \dot\theta_k^l\right)^2 \cos\left(\theta - \theta_h^l - \theta_k^l\right) + \left(\dot\theta - \dot\theta_h^r - \dot\theta_k^r\right)^2 \cos(\theta - \theta_h^r - \theta_k^r) \right\} \\
& -(m_1 L_3 + m_2 L_3 + m_3 d_3) \left\{ \left(\dot\theta - \dot\theta_h^l\right)^2 \cos\left(\theta - \theta_h^l\right) + \left(\dot\theta - \dot\theta_h^r\right)^2 \cos(\theta - \theta_h^r) \right\} \\
& +2(m_1 + m_2 + m_3)(d_4 - L_4)\dot\theta^2 \cos(\theta) \\
b_3 \;=\;& -m_1 d_1 \left\{ \left(\dot\theta - \dot\theta_h^l - \dot\theta_k^l - \dot\theta_a^l\right)^2 \sin\left(\theta - \theta_h^l - \theta_k^l - \theta_a^l\right) + \left(\dot\theta - \dot\theta_h^r - \dot\theta_k^r - \dot\theta_a^r\right)^2 \sin(\theta - \theta_h^r - \theta_k^r - \theta_a^r) \right\} \\
& -(m_1 L_2 + m_2 d_2) \left\{ \left(\dot\theta - \dot\theta_h^l - \dot\theta_k^l\right)^2 \sin\left(\theta - \theta_h^l - \theta_k^l\right) + \left(\dot\theta - \dot\theta_h^r - \dot\theta_k^r\right)^2 \sin(\theta - \theta_h^r - \theta_k^r) \right\} \\
& -(m_1 L_3 + m_2 L_3 + m_3 d_3) \left\{ \left(\dot\theta - \dot\theta_h^l\right)^2 \sin\left(\theta - \theta_h^l\right) + \left(\dot\theta - \dot\theta_h^r\right)^2 \sin(\theta - \theta_h^r) \right\} \\
& +2(m_1 + m_2 + m_3)(d_4 - L_4)\dot\theta^2 \sin(\theta) \\
b_4 \;=\;& -m_1 d_1 L_2 \left(\dot\theta - \dot\theta_h^l - \dot\theta_k^l\right)^2 \sin\left(-\theta_a^l\right) \\
& -m_1 d_1 L_3 \left(\dot\theta - \dot\theta_h^l\right)^2 \sin\left(-\theta_k^l - \theta_a^l\right) \\
& +m_1 d_1 (d_4 - L_4)\dot\theta^2 \sin\left(-\theta_h^l - \theta_k^l - \theta_a^l\right) \\
b_5 \;=\;& -m_1 d_1 L_2 \left\{ \left(\dot\theta - \dot\theta_h^l - \dot\theta_k^l\right)^2 - \left(\dot\theta - \dot\theta_h^l - \dot\theta_k^l - \dot\theta_a^l\right)^2 \right\} \sin\left(-\theta_a^l\right) \\
& -(m_2 d_2 + m_1 L_2) L_3 \left(\dot\theta - \dot\theta_h^l\right)^2 \sin\left(-\theta_k^l\right) \\
& +(m_2 d_2 + m_1 L_2)(d_4 - L_4)\dot\theta^2 \sin\left(-\theta_h^l - \theta_k^l\right) \\
& -m_1 d_1 L_3 \left(\dot\theta - \dot\theta_h^l\right)^2 \sin\left(-\theta_k^l - \theta_a^l\right) \\
& +m_1 d_1 (d_4 - L_4)\dot\theta^2 \sin\left(-\theta_h^l - \theta_k^l - \theta_a^l\right) \\
b_6 \;=\;& -m_1 d_1 L_3 \left\{ \left(\dot\theta - \dot\theta_h^l\right)^2 - \left(\dot\theta - \dot\theta_h^l - \dot\theta_k^l - \dot\theta_a^l\right)^2 \right\} \sin\left(-\theta_k^l - \theta_a^l\right) \\
& -(m_1 L_2 + m_2 d_2) L_3 \left\{ \left(\dot\theta - \dot\theta_h^l\right)^2 - \left(\dot\theta - \dot\theta_h^l - \dot\theta_k^l\right)^2 \right\} \sin\left(-\theta_k^l\right) \\
& +(m_1 L_3 + m_2 L_3 + m_3 d_3)(d_4 - L_4)\dot\theta^2 \sin\left(-\theta_h^l\right) \\
& -m_1 d_1 L_2 \left\{ \left(\dot\theta - \dot\theta_h^l - \dot\theta_k^l\right)^2 - \left(\dot\theta - \dot\theta_h^l - \dot\theta_k^l - \dot\theta_a^l\right)^2 \right\} \sin\left(-\theta_a^l\right) \\
& +(m_2 d_2 + m_1 L_2)(d_4 - L_4)\dot\theta^2 \sin\left(-\theta_h^l - \theta_k^l\right) \\
& +m_1 d_1 (d_4 - L_4)\dot\theta^2 \sin\left(-\theta_h^l - \theta_k^l - \theta_a^l\right) \\
b_7 \;=\;& -m_1 d_1 L_2 \left(\dot\theta - \dot\theta_h^r - \dot\theta_k^r\right)^2 \sin(-\theta_a^r) \\
& -m_1 d_1 L_3 \left(\dot\theta - \dot\theta_h^r\right)^2 \sin(-\theta_k^r - \theta_a^r) \\
& +m_1 d_1 (d_4 - L_4)\dot\theta^2 \sin(-\theta_h^r - \theta_k^r - \theta_a^r)
\end{aligned}
$$

$$
\begin{aligned}
b_8 &= -m_1d_1L_2\left\{\left(\dot\theta-\dot\theta_h^r-\dot\theta_k^r\right)^2-\left(\dot\theta-\dot\theta_h^r-\dot\theta_k^r-\dot\theta_a^r\right)^2\right\}\sin(-\theta_a^r)\\
&\quad-(m_2d_2+m_1L_2)L_3\left(\dot\theta-\dot\theta_h^r\right)^2\sin(-\theta_k^r)\\
&\quad+(m_2d_2+m_1L_2)(d_4-L_4)\dot\theta^2\sin(-\theta_h^r-\theta_k^r)\\
&\quad-m_1d_1L_3\left(\dot\theta-\dot\theta_h^r\right)^2\sin(-\theta_k^r-\theta_a^r)\\
&\quad+m_1d_1(d_4-L_4)\dot\theta^2\sin(-\theta_h^r-\theta_k^r-\theta_a^r)\\
b_9 &= -m_1d_1L_3\left\{\left(\dot\theta-\dot\theta_h^r\right)^2-\left(\dot\theta-\dot\theta_h^r-\dot\theta_k^r-\dot\theta_a^r\right)^2\right\}\sin(-\theta_k^r-\theta_a^r)\\
&\quad-(m_1L_2+m_2d_2)L_3\left\{\left(\dot\theta-\dot\theta_h^r\right)^2-\left(\dot\theta-\dot\theta_h^r-\dot\theta_k^r\right)^2\right\}\sin(-\theta_k^r)\\
&\quad+(m_1L_3+m_2L_3+m_3d_3)(d_4-L_4)\dot\theta^2\sin(-\theta_h^r)\\
&\quad-m_1d_1L_2\left\{\left(\dot\theta-\dot\theta_h^r-\dot\theta_k^r\right)^2-\left(\dot\theta-\dot\theta_h^r-\dot\theta_k^r-\dot\theta_a^r\right)^2\right\}\sin(-\theta_a^r)\\
&\quad+(m_2d_2+m_1L_2)(d_4-L_4)\dot\theta^2\sin(-\theta_h^r-\theta_k^r)\\
&\quad+m_1d_1(d_4-L_4)\dot\theta^2\sin(-\theta_h^r-\theta_k^r-\theta_a^r)\\
k_1 &= -2(m_1+m_2+m_3)(d_4-L_4)g\cos(\theta)\\
&\quad+(m_3d_3+m_1L_3+m_2L_3)g\left\{\cos\left(\theta-\theta_h^l\right)+\cos(\theta-\theta_h^r)\right\}\\
&\quad+(m_2d_2+m_1L_2)g\left\{\cos\left(\theta-\theta_h^l-\theta_k^l\right)+\cos(\theta-\theta_h^r-\theta_k^r)\right\}\\
&\quad+m_1d_1g\left\{\cos\left(\theta-\theta_h^l-\theta_k^l-\theta_a^l\right)+\cos(\theta-\theta_h^r-\theta_k^r-\theta_a^r)\right\}\\
k_2 &= 0\\
k_3 &= (2(m_1+m_2+m_3)+m_4)g\\
k_4 &= -m_1gd_1\cos\left(\theta-\theta_h^l-\theta_k^l-\theta_a^l\right)\\
k_5 &= -(m_2d_2+m_1L_2)g\cos\left(\theta-\theta_h^l-\theta_k^l\right)\\
&\quad-m_1gd_1\cos\left(\theta-\theta_h^l-\theta_k^l-\theta_a^l\right)\\
k_6 &= -(m_3d_3+m_1L_3+m_2L_3)g\cos\left(\theta-\theta_h^l\right)\\
&\quad-(m_2d_2+m_1L_2)g\cos\left(\theta-\theta_h^l-\theta_k^l\right)\\
&\quad-m_1gd_1\cos\left(\theta-\theta_h^l-\theta_k^l-\theta_a^l\right)\\
k_7 &= -m_1d_1g\cos(\theta-\theta_h^r-\theta_k^r-\theta_a^r)\\
k_8 &= -(m_2d_2+m_1L_2)g\cos(\theta-\theta_h^r-\theta_k^r)\\
&\quad-m_1d_1g\cos(\theta-\theta_h^r-\theta_k^r-\theta_a^r)\\
k_9 &= -(m_3d_3+m_1L_3+m_2L_3)g\cos(\theta-\theta_h^r)\\
&\quad-(m_2d_2+m_1L_2)g\cos(\theta-\theta_h^r-\theta_k^r)\\
&\quad-m_1d_1g\cos(\theta-\theta_h^r-\theta_k^r-\theta_a^r)\\
g_1 &= -F_{yrt}\left\{L_1\cos\left(\theta-\theta_h^l-\theta_k^l-\theta_a^l\right)+L_2\cos\left(\theta-\theta_h^l-\theta_k^l\right)\right.
\end{aligned}
$$

$$+L_3\cos\left(\theta-\theta_h^l\right)-(d_4-L_4)\cos(\theta)\Big\}$$

$$-F_{yrh}\Big\{L_0\cos\left(\left(\theta-\theta_h^l-\theta_k^l-\theta_a^l\right)-\phi\right)+L_2\cos\left(\theta-\theta_h^l-\theta_k^l\right)$$

$$+L_3\cos\left(\theta-\theta_h^l\right)-(d_4-L_4)\cos(\theta)\Big\}$$

$$-F_{ylt}\{L_1\cos(\theta-\theta_h^r-\theta_k^r-\theta_a^r)+L_2\cos(\theta-\theta_h^r-\theta_k^r)$$

$$+L_3\cos(\theta-\theta_h^r)-(d_4-L_4)\cos(\theta)\}$$

$$-F_{ylh}\{L_0\cos((\theta-\theta_h^r-\theta_k^r-\theta_a^r)-\phi)+L_2\cos(\theta-\theta_h^r-\theta_k^r)$$

$$+L_3\cos(\theta-\theta_h^r)-(d_4-L_4)\cos(\theta)\}$$

$$+F_{xrt}\Big\{L_1\sin\left(\theta-\theta_h^l-\theta_k^l-\theta_a^l\right)+L_2\sin\left(\theta-\theta_h^l-\theta_k^l\right)$$

$$+L_3\sin\left(\theta-\theta_h^l\right)-(d_4-L_4)\sin(\theta)\Big\}$$

$$+F_{xrh}\Big\{L_0\sin\left(\left(\theta-\theta_h^l-\theta_k^l-\theta_a^l\right)-\phi\right)+L_2\sin\left(\theta-\theta_h^l-\theta_k^l\right)$$

$$+L_3\sin\left(\theta-\theta_h^l\right)-(d_4-L_4)\sin(\theta)\Big\}$$

$$+F_{xlt}\{L_1\sin(\theta-\theta_h^r-\theta_k^r-\theta_a^r)+L_2\sin(\theta-\theta_h^r-\theta_k^r)$$

$$+L_3\sin(\theta-\theta_h^r)-(d_4-L_4)\sin(\theta)\}$$

$$+F_{xlh}\{L_0\sin((\theta-\theta_h^r-\theta_k^r-\theta_a^r)-\phi)+L_2\sin(\theta-\theta_h^r-\theta_k^r)$$

$$+L_3\sin(\theta-\theta_h^r)-(d_4-L_4)\sin(\theta)\}$$

$$g_2 = -F_{xrt}-F_{xrh}-F_{xlt}-F_{xlh}$$

$$g_3 = -F_{yrt}-F_{yrh}-F_{ylt}-F_{ylh}$$

$$g_4 = F_{yrt}L_1\cos\left(\theta-\theta_h^l-\theta_k^l-\theta_a^l\right)$$

$$-F_{xrt}L_1\sin\left(\theta-\theta_h^l-\theta_k^l-\theta_a^l\right)$$

$$+F_{yrh}L_0\cos\left(\left(\theta-\theta_h^l-\theta_k^l-\theta_a^l\right)-\phi\right)$$

$$-F_{xrh}L_0\sin\left(\left(\theta-\theta_h^l-\theta_k^l-\theta_a^l\right)-\phi\right)$$

$$g_5 = F_{yrt}\Big\{L_2\cos\left(\theta-\theta_h^l-\theta_k^l\right)+L_1\cos\left(\theta-\theta_h^l-\theta_k^l-\theta_a^l\right)\Big\}$$

$$+F_{yrh}\Big\{L_2\cos\left(\theta-\theta_h^l-\theta_k^l\right)+L_0\cos\left(\left(\theta-\theta_h^l-\theta_k^l-\theta_a^l\right)-\phi\right)\Big\}$$

$$-F_{xrt}\Big\{L_2\sin\left(\theta-\theta_h^l-\theta_k^l\right)+L_1\sin\left(\theta-\theta_h^l-\theta_k^l-\theta_a^l\right)\Big\}$$

$$-F_{xrh}\Big\{L_2\sin\left(\theta-\theta_h^l-\theta_k^l\right)+L_0\sin\left(\left(\theta-\theta_h^l-\theta_k^l-\theta_a^l\right)-\phi\right)\Big\}$$

$$g_6 = F_{yrt}\Big\{L_3\cos\left(\theta-\theta_h^l\right)+L_2\cos\left(\theta-\theta_h^l-\theta_k^l\right)+L_1\cos\left(\theta-\theta_h^l-\theta_k^l-\theta_a^l\right)\Big\}$$

$$+F_{yrh}\Big\{L_3\cos\left(\theta-\theta_h^l\right)+L_2\cos\left(\theta-\theta_h^l-\theta_k^l\right)+L_0\cos\left(\left(\theta-\theta_h^l-\theta_k^l-\theta_a^l\right)-\phi\right)\Big\}$$

$$-F_{xrt}\Big\{L_3\sin\left(\theta-\theta_h^l\right)+L_2\sin\left(\theta-\theta_h^l-\theta_k^l\right)+L_1\sin\left(\theta-\theta_h^l-\theta_k^l-\theta_a^l\right)\Big\}$$

$$-F_{xrh}\Big\{L_3\sin\left(\theta-\theta_h^l\right)+L_2\sin\left(\theta-\theta_h^l-\theta_k^l\right)+L_0\sin\left(\left(\theta-\theta_h^l-\theta_k^l-\theta_a^l\right)-\phi\right)\Big\}$$

$$g_7 = F_{ylt}L_1\cos(\theta-\theta_h^r-\theta_k^r-\theta_a^r)$$

$$-F_{xlt}L_1\sin(\theta-\theta_h^r-\theta_k^r-\theta_a^r)$$

$$
\begin{aligned}
& +F_{ylh}L_0\cos((\theta-\theta_h^r-\theta_k^r-\theta_a^r)-\phi) \\
& -F_{xlh}L_0\sin((\theta-\theta_h^r-\theta_k^r-\theta_a^r)-\phi) \\
g_8 \;=\; & F_{ylt}\{L_2\cos(\theta-\theta_h^r-\theta_k^r)+L_1\cos(\theta-\theta_h^r-\theta_k^r-\theta_a^r)\} \\
& +F_{ylh}\{L_2\cos(\theta-\theta_h^r-\theta_k^r)+L_0\cos((\theta-\theta_h^r-\theta_k^r-\theta_a^r)-\phi)\} \\
& -F_{xlt}\{L_2\sin(\theta-\theta_h^r-\theta_k^r)+L_1\sin(\theta-\theta_h^r-\theta_k^r-\theta_a^r)\} \\
& -F_{xlh}\{L_2\sin(\theta-\theta_h^r-\theta_k^r)+L_0\sin((\theta-\theta_h^r-\theta_k^r-\theta_a^r)-\phi)\} \\
g_9 \;=\; & F_{ylt}\{L_3\cos(\theta-\theta_h^r)+L_2\cos(\theta-\theta_h^r-\theta_k^r)+L_1\cos(\theta-\theta_h^r-\theta_k^r-\theta_a^r)\} \\
& +F_{ylh}\{L_3\cos(\theta-\theta_h^r)+L_2\cos(\theta-\theta_h^r-\theta_k^r)+L_0\cos((\theta-\theta_h^r-\theta_k^r-\theta_a^r)-\phi)\} \\
& -F_{xlt}\{L_3\sin(\theta-\theta_h^r)+L_2\sin(\theta-\theta_h^r-\theta_k^r)+L_1\sin(\theta-\theta_h^r-\theta_k^r-\theta_a^r)\} \\
& -F_{xlh}\{L_3\sin(\theta-\theta_h^r)+L_2\sin(\theta-\theta_h^r-\theta_k^r)+L_0\sin((\theta-\theta_h^r-\theta_k^r-\theta_a^r)-\phi)\}
\end{aligned}
$$

A.2 The model of ground reaction forces

The ground reaction force is modeled using nonlinear dampers and springs. By defining the sagittal positions of left-toe, left-heel, right-toe and right-heel, respectively, as $(X^{l,t},Y^{l,t})$, $(X^{l,h},Y^{l,h})$, $(X^{r,t},Y^{r,t})$, $(X^{r,h},Y^{r,h})$, the vertical ground reaction forces acting at these four points in this order are defined as follows.

$$
\begin{aligned}
F_{ylt} &= \begin{cases} -\kappa Y^{l,t}-\lambda_v\dot{Y}^{l,t}, & \text{if } Y^{l,t}<0 \text{ and } -\kappa Y^{l,t}-\lambda_v\dot{Y}^{r,t}>0 \\ 0, & \text{otherwise} \end{cases} \\
F_{ylh} &= \begin{cases} -\kappa Y^{l,h}-\lambda_v\dot{Y}^{l,h}, & \text{if } Y^{l,h}<0 \text{ and } -\kappa Y^{l,h}-\lambda_v\dot{Y}^{l,h}>0 \\ 0, & \text{otherwise} \end{cases} \\
F_{yrt} &= \begin{cases} -\kappa Y^{r,t}-\lambda_v\dot{Y}^{r,t}, & \text{if } Y^{r,t}<0 \text{ and } -\kappa Y^{r,t}-\lambda_v\dot{Y}^{r,t}>0 \\ 0, & \text{otherwise} \end{cases} \\
F_{yrh} &= \begin{cases} -\kappa Y^{r,h}-\lambda_v\dot{Y}^{r,h}, & \text{if } Y^{r,h}<0 \text{ and } -\kappa Y^{r,h}-\lambda_v\dot{Y}^{r,h}>0 \\ 0, & \text{otherwise} \end{cases}
\end{aligned}
$$

The horizontal ground reaction forces acting at the four points in the order of listed above are defined as follows.

$$
\begin{aligned}
F_{xlt} &= -\frac{2\nu F_{ylt}}{\pi}\tan^{-1}\left(\frac{\lambda_h\pi\dot{X}^{l,t}}{2\nu F_{ylt}}\right) \\
F_{xlh} &= -\frac{2\nu F_{ylh}}{\pi}\tan^{-1}\left(\frac{\lambda_h\pi\dot{X}^{l,h}}{2\nu F_{ylh}}\right) \\
F_{xrt} &= -\frac{2\nu F_{yrt}}{\pi}\tan^{-1}\left(\frac{\lambda_h\pi\dot{X}^{r,t}}{2\nu F_{yrt}}\right) \\
F_{xrh} &= -\frac{2\nu F_{yrh}}{\pi}\tan^{-1}\left(\frac{\lambda_h\pi\dot{X}^{r,h}}{2\nu F_{yrh}}\right)
\end{aligned}
$$

The parameters used in the model of ground reaction forces are summarized in the following Table.

B Numerical evaluation of Jacobian matrix and Floquet multipliers

The state space representation of the biped model as a non-autonomous dynamical system with the vector field $f(x,t)$ is defined by Eq. 10 in the main text, and its the Jacobian $D_{\phi_0}(t)$ for its linearized equation

Symbol	Description	
κ	Ground reaction force parameter	20000
λ_v	Ground reaction force parameter	300
λ_h	Ground reaction force parameter	2000
ν	Ground reaction force parameter	0.3

Table 1: Parameter values used in the model of ground reaction forces

is defined by Eq. 14. The numerical evaluation of $D_{\phi_0}(t)$ was performed by numerical partial derivative of $f(x,t)$ using the double side finite difference method. That is, the i-j element of $D_{\phi_0}(t)$ was obtained as

$$\frac{\partial f_i}{\partial x_j}(x_r(t)){\sim}\frac{f_i(x_r(t){+}\Delta x_j,t){-}f_i(x_r(t){-}\Delta x_j,t)}{2\Delta x_j}$$

where $i{=}1,\cdots,19$, and Δx_j is the difference of the i-th element of the state vector x. Throughout this study, we decided to use $\Delta x_j{=}10^{-3}$. The size of Δx_j should be determined with a care, because it should balance with the time step Δt, which was 10^{-5} in this study. Our choice of Δx_j was based on the fact that changes in $x_r(t)$ for the short duration of time Δt along the one gait cycle is about between 10^{-3} and 10^{-6}. Hence the value of Δx_j was the lower band of this variation.

We validated the use of $\Delta x_j{=}10^{-3}$ by examining that the numerical evaluation of $D_{\phi_0}(t)$ using $\Delta x_j{=}$ 10^{-3} and loci of FMs as the function of the PD-gains, as in Fig.4 of the main text, for various values of Δx_j ranging from 10^{-1} to 10^{-6}. The result of this examination showed that was the loci of FMs were qualitatively and quantitatively the same quite robust for a wide range of Δx_j between 10^{-2} and $5{\times}10^{-6}$, and $\Delta x_j{=}10^{-3}$ is the middle of this valid range.

C Impedance and dynamic impedance

Here we summarize several definitions of dynamic impedance (stiffness and viscosity). Remind the biped motion equation as

$$J(q)\ddot{q}{+}B(q,\omega){+}K(q){+}G(q,\omega){=}U_{\text{ff}}(\bar{q}(t),\dot{\bar{q}}(t),\ddot{\bar{q}}(t)){+}U_{\text{fb}}(q,\omega,\bar{q}(t),\dot{\bar{q}}(t)) \tag{1}$$

where

$$U_{\text{fb}}{=}P(\bar{q}(t){-}q){+}D(\dot{\bar{q}}(t){-}\omega) \tag{2}$$

with

$$P{=}\text{diag}\{0,0,0,P_a,P_k,P_h,P_a,P_k,P_h\}$$
$$D{=}\text{diag}\{0,0,0,D_a,D_k,D_h,D_a,D_k,D_h\},$$

and denote the total joint torque as $U{=}U_{\text{ff}}{+}U_{\text{fb}}$. The joint stiffness K_d and viscosity B_d are usually defined, respectively, by the derivatives of the total joint torque with respect to the position and the velocity, which are equal to P and D in our biped model. That is,

$$\begin{aligned} K_d &\equiv -\frac{\partial U}{\partial q} = P, \\ B_d &\equiv -\frac{\partial U}{\partial \omega} = D. \end{aligned} \tag{3}$$

Thus, we considered simply the PD-gains of the feedback controller as the joint impedance in this study.

It is also worthwhile to consider a different type of joint impedance, we call it total impedance, which is more directly related to stability of the steady state solution $\bar{q}$,$\dot{\bar{q}}$ as the limit cycle. Considering a perturbed solution as $q{=}\bar{q}{+}\tilde{q}$, $\omega{=}\dot{\bar{q}}{+}\tilde{\omega}$, $\ddot{q}{=}\ddot{\bar{q}}{+}\ddot{\tilde{q}}$. We have

$$J(\bar{q}{+}\tilde{q})(\ddot{\bar{q}}{+}\ddot{\tilde{q}}){+}B(\bar{q}{+}\tilde{q},\dot{\bar{q}}{+}\tilde{\omega}){+}K(\bar{q}{+}\tilde{q}){+}G(\bar{q}{+}\tilde{q},\dot{\bar{q}}{+}\tilde{\omega}){=}U(\bar{q}{+}\tilde{q},\dot{\bar{q}}{+}\tilde{\omega},\bar{q},\dot{\bar{q}}),$$

Using first order Taylor expansion,

$$\begin{aligned}&\left[J(\bar{q}){+}\left\{\tfrac{\partial J}{\partial q}(\bar{q}),\tilde{q}\right\}\right](\ddot{\bar{q}}{+}\ddot{\tilde{q}}){+}B(\bar{q},\dot{\bar{q}}){+}\tfrac{\partial B}{\partial q}(\bar{q},\dot{\bar{q}})\tilde{q}{+}\tfrac{\partial B}{\partial \omega}(\bar{q},\dot{\bar{q}})\tilde{\omega}\\ &{+}K(\bar{q}){+}\tfrac{\partial K}{\partial q}(\bar{q})\tilde{q}{+}G(\bar{q},\dot{\bar{q}}){+}\tfrac{\partial G}{\partial q}(\bar{q},\dot{\bar{q}})\tilde{q}{+}\tfrac{\partial G}{\partial \omega}(\bar{q},\dot{\bar{q}})\tilde{\omega}\\ &{=}U(\bar{q},\dot{\bar{q}}){+}\tfrac{\partial U}{\partial q}(\bar{q},\dot{\bar{q}})\tilde{q}{+}\tfrac{\partial U}{\partial \omega}(\bar{q},\dot{\bar{q}})\tilde{\omega}\end{aligned}$$

where

$$\left\{\frac{\partial J}{\partial q}(\bar{q}),\Delta q\right\}{\equiv}\sum_{i=1}^{9}\frac{\partial J}{\partial q_i}(\bar{q})\tilde{q}_i.$$

By neglecting the second and higher order terms, with the consideration of Eq.1, this can be simplified as

$$\begin{aligned}J(\bar{q})\ddot{\tilde{q}}{+}\left\{\frac{\partial J}{\partial q}(\bar{q}),\tilde{q}\right\}\ddot{\bar{q}}{+}\frac{\partial B}{\partial q}(\bar{q},\dot{\bar{q}})\tilde{q}{+}\frac{\partial B}{\partial \omega}(\bar{q},\dot{\bar{q}})\tilde{\omega}&\\ {+}\frac{\partial K}{\partial q}(\bar{q})\tilde{q}{+}\frac{\partial G}{\partial q}(\bar{q},\dot{\bar{q}})\tilde{q}{+}\frac{\partial G}{\partial \omega}(\bar{q},\dot{\bar{q}})\tilde{\omega}&\\ {=}\frac{\partial U}{\partial q}(\bar{q},\dot{\bar{q}})\tilde{q}{+}\frac{\partial U}{\partial \omega}(\bar{q},\dot{\bar{q}})\tilde{\omega}.&\end{aligned}\tag{4}$$

where the second term of the left-hand side is defined as

$$\begin{aligned}\left\{\frac{\partial J}{\partial q}(\bar{q}),\tilde{q}\right\}\ddot{\bar{q}} &\equiv \left(\frac{\partial J}{\partial q_1}(\bar{q})\tilde{q}_1{+}\frac{\partial J}{\partial q_2}(\bar{q})\tilde{q}_2{+}\frac{\partial J}{\partial q_3}(\bar{q})\tilde{q}_3{+}\cdots{+}\frac{\partial J}{\partial q_9}(\bar{q})\tilde{q}_9\right)\ddot{\bar{q}}\\ &= \frac{\partial J}{\partial q_1}(\bar{q})\tilde{q}_1\ddot{\bar{q}}{+}\frac{\partial J}{\partial q_2}(\bar{q})\tilde{q}_2\ddot{\bar{q}}{+}\frac{\partial J}{\partial q_3}(\bar{q})\tilde{q}_3\ddot{\bar{q}}{+}\cdots{+}\frac{\partial J}{\partial q_9}(\bar{q})\tilde{q}_9\ddot{\bar{q}}\\ &= \frac{\partial J}{\partial q_1}(\bar{q})\ddot{\bar{q}}\tilde{q}_1{+}\frac{\partial J}{\partial q_2}(\bar{q})\ddot{\bar{q}}\tilde{q}_2{+}\frac{\partial J}{\partial q_3}(\bar{q})\ddot{\bar{q}}\tilde{q}_3{+}\cdots{+}\frac{\partial J}{\partial q_9}(\bar{q})\ddot{\bar{q}}\tilde{q}_9\\ &= \left[\frac{\partial J}{\partial q_1}(\bar{q})\ddot{\bar{q}}\ \ \frac{\partial J}{\partial q_2}(\bar{q})\ddot{\bar{q}}\ \ \frac{\partial J}{\partial q_3}(\bar{q})\ddot{\bar{q}}\ \ \cdots\ \ \frac{\partial J}{\partial q_9}(\bar{q})\ddot{\bar{q}}\right]\tilde{q}\\ &= \frac{\partial J}{\partial q}\ddot{\bar{q}}\ \tilde{q}.\end{aligned}\tag{5}$$

Note that, in the last line of this equation, we used the following notation:

$$\frac{\partial J}{\partial q}\ddot{\bar{q}}{\equiv}\left[\frac{\partial J}{\partial q_1}(\bar{q})\ddot{\bar{q}}\ \ \frac{\partial J}{\partial q_2}(\bar{q})\ddot{\bar{q}}\ \ \frac{\partial J}{\partial q_3}(\bar{q})\ddot{\bar{q}}\ \ \cdots\ \ \frac{\partial J}{\partial q_9}(\bar{q})\ddot{\bar{q}}\right].$$

Collecting the terms with respect to $\tilde{q}$ and its derivatives, we have the following linearized equation, which describes the dynamic evolution of perturbation, in another phrase, error dynamics around the limit cycle.

$$J(q)\ddot{\tilde{q}}{+}B_{\text{total}}\tilde{\omega}{+}K_{\text{total}}\tilde{q}{=}0\tag{6}$$

where

$$K_{\text{total}} \equiv -\frac{\partial U}{\partial q}(\bar{q},\dot{\bar{q}}) + \frac{\partial B}{\partial q}(\bar{q},\dot{\bar{q}}) + \frac{\partial K}{\partial q}(\bar{q}) + \frac{\partial G}{\partial q}(\bar{q},\dot{\bar{q}}) + \frac{\partial J}{\partial q}\ddot{\bar{q}}, \tag{7}$$

and

$$B_{\text{total}} = -\frac{\partial U}{\partial \omega}(\bar{q},\dot{\bar{q}}) + \frac{\partial B}{\partial \omega}(\bar{q},\dot{\bar{q}}) + \frac{\partial G}{\partial \omega}(\bar{q},\dot{\bar{q}}). \tag{8}$$

We call K_{total} and B_{total} total stiffness and total dynamic viscosity, respectively. This can also be interpreted as the dynamic balance on each timing on the limit cycle.

$$J(q)\ddot{\tilde{q}} = -B_{\text{total}}\tilde{\omega} - K_{\text{total}}\tilde{q} \tag{9}$$

In Eq.9, if the perturbation intends to cause the deviation away from the limit cycle, K_{total} and B_{total} will counteract the diverging torque and drive the trajectory back to the limit cycle. So it is also natural to define K_{total} and B_{total} as joint stiffness and viscosity.

Furthermore, K_{total} and B_{total} can be conveniently related to the Jacobian matrix around limit cycle solution. This is because of the following derivation. The state space representation is also rewritten here.

$$\frac{d}{dt}\begin{pmatrix} q \\ \omega \end{pmatrix} = \begin{pmatrix} \omega \\ J^{-1}(q)(U(q,\omega,\bar{q},\dot{\bar{q}}) - B(q,\omega) - K(q) - G(q,\omega)) \end{pmatrix} \equiv \begin{pmatrix} F_1(\omega) \\ F_2(q,\omega,U) \end{pmatrix}. \tag{10}$$

Jacobian matrix could be obtained by differentiating the vector field of Eq.10 as follows:

$$\begin{aligned}
\frac{\partial F_1}{\partial q} &= O \\
\frac{\partial F_1}{\partial \omega} &= I \\
\frac{\partial F_2}{\partial q} &= \frac{\partial J^{-1}}{\partial q}(U-B-K-G) + J^{-1}\left(\frac{\partial U}{\partial q} - \frac{\partial B}{\partial q} - \frac{\partial K}{\partial q} - \frac{\partial G}{\partial q}\right) \\
&= -J^{-1}\frac{\partial J}{\partial q}J^{-1}(U-B-K-G) + J^{-1}\left(\frac{\partial U}{\partial q} - \frac{\partial B}{\partial q} - \frac{\partial K}{\partial q} - \frac{\partial G}{\partial q}\right) \\
&= -J^{-1}\frac{\partial J}{\partial q}\ddot{q} + J^{-1}\left(\frac{\partial U}{\partial q} - \frac{\partial B}{\partial q} - \frac{\partial K}{\partial q} - \frac{\partial G}{\partial q}\right) \\
&= J^{-1}\left(\frac{\partial U}{\partial q} - \frac{\partial B}{\partial q} - \frac{\partial K}{\partial q} - \frac{\partial G}{\partial q} - \frac{\partial J}{\partial q}\ddot{q}\right) \\
\frac{\partial F_2}{\partial \omega} &= J^{-1}\left(\frac{\partial U}{\partial \omega} - \frac{\partial B}{\partial \omega} - \frac{\partial G}{\partial \omega}\right)
\end{aligned} \tag{11}$$

For the Jacobian matrix evaluated around the limit cycle, which is denoted by D_{ϕ_0} in the main text, from the comparison, we can easily see that

$$\frac{\partial F_2}{\partial q} = -J^{-1}K_{\text{total}}$$

$$\frac{\partial F_2}{\partial \omega} = -J^{-1}B_{\text{total}}$$

Thus D_{ϕ_0} can be written as

$$D_{\phi_0}(t) = \begin{pmatrix} \frac{\partial F_1}{\partial q} & \frac{\partial F_1}{\partial \omega} \\ \frac{\partial F_2}{\partial q} & \frac{\partial F_2}{\partial \omega} \end{pmatrix} = \begin{pmatrix} 0 & I \\ -J^{-1}K_{\text{total}} & -J^{-1}B_{\text{total}} \end{pmatrix} \tag{12}$$

In the calculation of Floquet matrix, the $D_{\phi_0}(t)$ has already been calculated numerically. So it is very prompt to obtain the dynamic stiffness and dynamic viscosity by multiply $-J$ to $\partial F_2/\partial q$ and $\partial F_2/\partial \omega$ block of D_{ϕ_0}. If we only care about the leg joints and are not interested in the correlated influence between the joints, we select only the diagonals of K_{total} and B_{total}, and consider them as dynamic stiffness and dynamic viscosity of leg joint. The non-diagonal element could be interpreted as inter-joint dynamic impedance. Dynamic stiffness and dynamic viscosity of leg joint during steady-state gait with the feedback PD gains of 1500 and 10 are illustrated in Fig.1 and Fig.2. It is noted that the peaks are due to the foot impact.

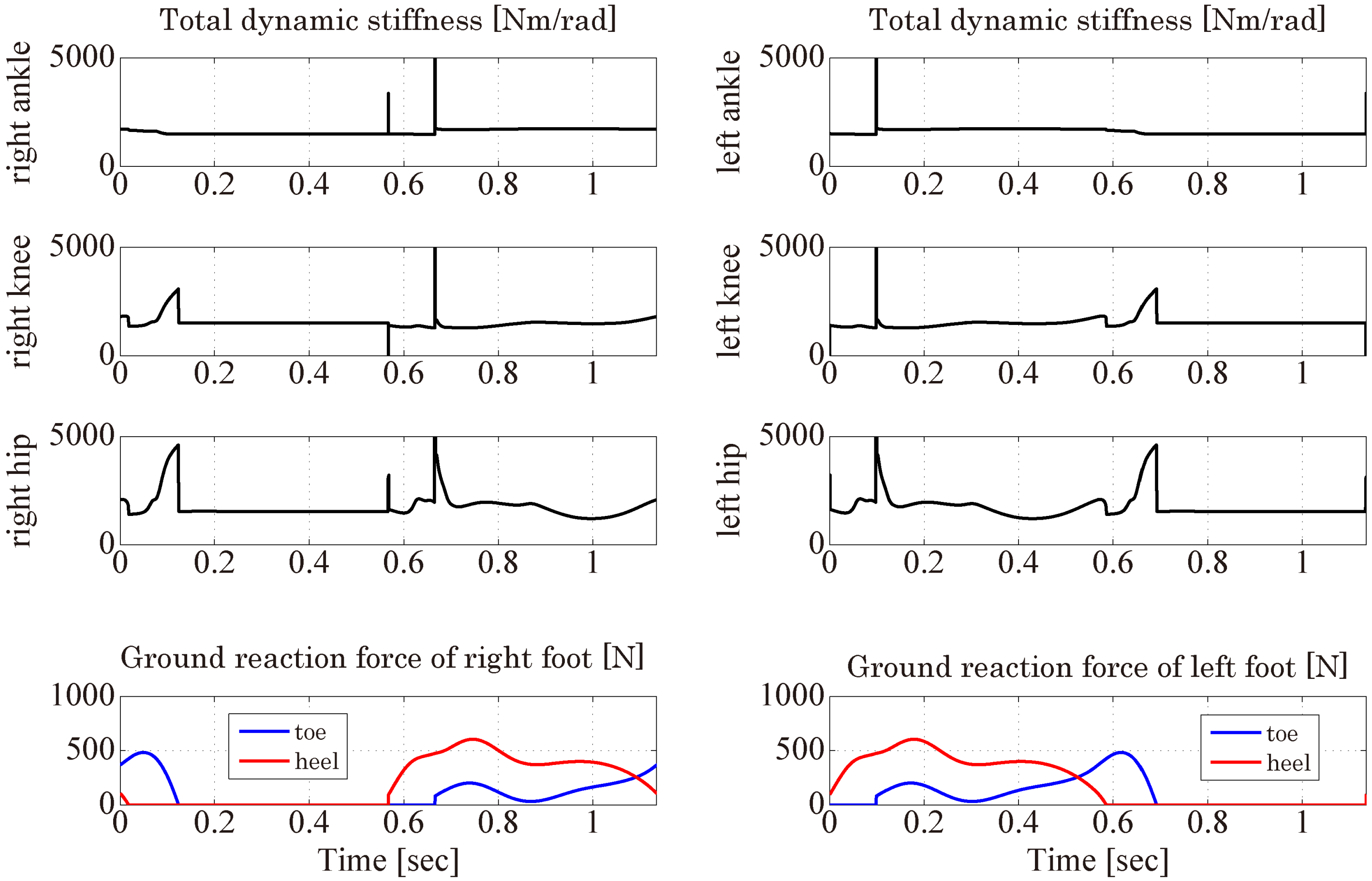

Figure 1: Dynamic total stiffness and viscosity during one gait cycle

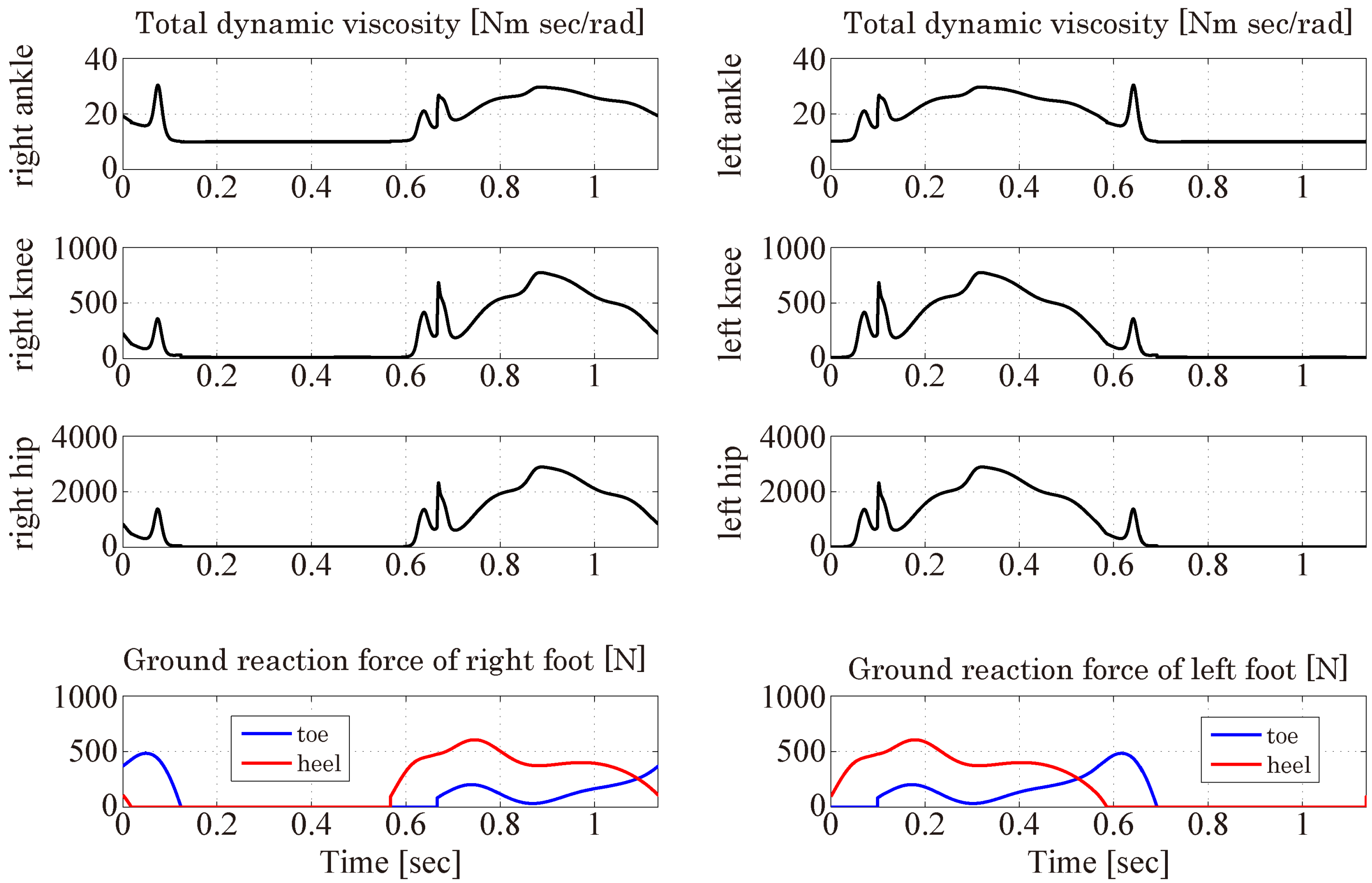

Figure 2: Dynamic total viscosity during one gait cycle
